# EphA3 Expressed in the Chicken Tectum Stimulates Nasal Retinal Ganglion Cell Axon Growth and Is Required for Retinotectal Topographic Map Formation

**DOI:** 10.1371/journal.pone.0038566

**Published:** 2012-06-07

**Authors:** Ana Laura Ortalli, Luciano Fiore, Jennifer Di Napoli, Melina Rapacioli, Marcelo Salierno, Roberto Etchenique, Vladimir Flores, Viviana Sanchez, Néstor Gabriel Carri, Gabriel Scicolone

**Affiliations:** 1 Laboratory of Developmental Neurobiology, Institute of Cell Biology and Neurosciences “Prof. E. De Robertis” (UBA-CONICET), School of Medicine, University of Buenos Aires, Buenos Aires, Argentina; 2 Institute of Multidisciplinary Cell Biology (CONICET-CIC), La Plata, Argentina; 3 Interdisciplinary Group in Theoretical Biology, Department of Bioestructural Sciences, Favaloro University, Buenos Aires, Argentina; 4 Department of Inorganic, Analytical and Physical Chemistry (INQUIMAE), Faculty of Exact and Natural Sciences, University of Buenos Aires, Buenos Aires, Argentina; Tokyo Medical and Dental University, Japan

## Abstract

**Background:**

Retinotopic projection onto the tectum/colliculus constitutes the most studied model of topographic mapping and Eph receptors and their ligands, the ephrins, are the best characterized molecular system involved in this process. Ephrin-As, expressed in an increasing rostro-caudal gradient in the tectum/colliculus, repel temporal retinal ganglion cell (RGC) axons from the caudal tectum and inhibit their branching posterior to their termination zones. However, there are conflicting data regarding the nature of the second force that guides nasal axons to invade and branch only in the caudal tectum/colliculus. The predominant model postulates that this second force is produced by a decreasing rostro-caudal gradient of EphA7 which repels nasal optic fibers and prevents their branching in the rostral tectum/colliculus. However, as optic fibers invade the tectum/colliculus growing throughout this gradient, this model cannot explain how the axons grow throughout this repellent molecule.

**Methodology/Principal Findings:**

By using chicken retinal cultures we showed that EphA3 ectodomain stimulates nasal RGC axon growth in a concentration dependent way. Moreover, we showed that nasal axons choose growing on EphA3-expressing cells and that EphA3 diminishes the density of interstitial filopodia in nasal RGC axons. Accordingly, *in vivo* EphA3 ectodomain misexpression directs nasal optic fibers toward the caudal tectum preventing their branching in the rostral tectum.

**Conclusions:**

We demonstrated *in vitro* and *in vivo* that EphA3 ectodomain (which is expressed in a decreasing rostro-caudal gradient in the tectum) is necessary for topographic mapping by stimulating the nasal axon growth toward the caudal tectum and inhibiting their branching in the rostral tectum. Furthermore, the ability of EphA3 of stimulating axon growth allows understanding how optic fibers invade the tectum growing throughout this molecular gradient. Therefore, opposing tectal gradients of repellent ephrin-As and of axon growth stimulating EphA3 complement each other to map optic fibers along the rostro-caudal tectal axis.

## Introduction

Nervous system functions depend upon precisely organized neuronal connections. Many axons establish an ordered arrangement in their target in such way that neighbouring cells project to neighbouring parts in the target forming a topographic map [1]. The main model to study the development of topographic maps is the retinal ganglion cell (RGC) projection to the optic tectum or superior colliculus, which is organized in two orthogonally oriented axes. Nasal RGCs project to the caudal tectum and temporal RGCs project to the rostral tectum, whereas dorsal RGCs project to the ventral tectum and ventral RGCs project to the dorsal tectum [1], [2]. RGC axons invade the chicken tectum from the rostral pole and follow its developmental gradient axis toward the caudal pole [1], [3], [4], [5]. These axons overshoot their future target areas along the rostro-caudal axis but form branches around the position of their future termination zones, which are formed by the arborization of the appropriately located branches and the pruning of the overshooting axonal leading tips [2], [6]. The branches invade deeper retino-recipient layers, where they establish synaptic connections [2], [3], [4], [5].

The molecular mechanisms involved in topographic mapping agree with Sperry's theory of chemoaffinity. Sperry predicted that RGC axons find their targets throughout interactions involving recognition molecules that are differentially expressed on their growth cones and on tectal cells. Furthermore, he proposed that each location in the tectum has a unique molecular address determined by the graded distribution of the topographic recognition molecules. Each RGC has a unique profile of receptors for those molecules, resulting in a position-dependent, differential response [1], [7]. It has been later proposed that activity-independent [8], [9], [10] and -dependent interaxonal competition [11], [12], [13] refines this topographic map.

Eph receptors and their ephrin ligands are expressed in gradients in both the retina and the tectum/colliculus, and several groups have shown that they represent the main molecular system controlling the mapping of retinal projections onto the tectum/colliculus [1], [2], [14], [15]. The Eph receptors are a family of widely expressed receptor tyrosine kinases comprising ten EphA and six EphB members. EphA and EphB receptors promiscuously bind the six glycosylphosphatidylinositol (GPI)-linked ephrin-A ligands and the three transmembrane ephrin-B ligands respectively. The fact that the ephrins are membrane-bound proteins allows the Eph-ephrin interaction to produce bidirectional signaling with morphologic consequences in both interacting cells [16], [17]. EphA receptors and ephrin-As define the topographic retinotectal/collicular connections along the rostro-caudal axis, whereas EphB receptors and ephrin-Bs have been found to be involved in guidance along the dorso-ventral axis [1], [2], [14], [15]. This is achieved through opposing gradients of Ephs and ephrins in both the retina and the tectum [1], [2], [14], [18], [19]. Thus, EphA3, A5 and A6 are expressed in an increasing naso-temporal gradient [18], [20], [21], whereas EphA4 presents an even distribution along the retina, with a decreasing naso-dorsal to temporo-ventral gradient of phosphorylation [18]. EphA3, A4, A7 and A8 are expressed in a decreasing rostro-caudal gradient in the tectum/colliculus [18], [22], [23], [24] while ephrin-A2, -A5 and –A6 are expressed in a decreasing naso-temporal retinal gradient [18], [19], [25] and in an increasing rostro-caudal tectal gradient [19], [23], [26].

Ephrin-A2 and ephrin-A5 expressed in the caudal tectum/colliculus are growth cone repellents [26], [27], [28], [29], [30] and interstitial branching inhibitors [6], [31] that preferentially affect temporal RGC axons by activating their EphA receptors [9], [20], [21], [32]. Thus, tectal ephrin-As prevent temporal RGC axons from branching caudally to their appropriate target area. It has been shown that ephrin-As of RGC axons diminish the repulsive response of axonal EphA receptors to tectal ephrin-As, preventing repulsion of nasal RGC axons from the caudal tectum [25], [33], [34]. However, these data do not explain why nasal RGC axons grow toward the caudal tectum without branching rostrally to their appropriate target area.

Thus, one gradient of activity per axis (tectal ephrin-As) is not sufficient to establish retinotopic connections, because a single cue gradient would cause all axons to migrate to and branch at one end of the map [2]. Instead, counterbalanced forces are thought to be required, with each axon branching where these opposing forces balance out [1], [35]. Conflicting models have been postulated about the second mapping force [1], [2], [35]. The most accepted model proposes that this second force is produced by a decreasing rostro-caudal gradient of EphA7 which repels nasal optic fibers and prevent them from branching in the rostral tectum/colliculus [1], [24], [36], [37], [38], [39]. However, as optic fibers invade the tectum/colliculus throughout the highest part of this gradient, this model cannot explain how the axons invade the tectum/colliculus without being repelled by EphA7 [1]. Moreover, the role of tectal EphAs has not been evaluated in non mammalian vertebrates throughout *in vivo* experiments. Although differences in retinotectal/collicular mapping have been mainly described between fishes and amphibian versus birds and mammals [1], [40], EphA3 and ephrin-A6 are only expressed in birds visual system and recent works suggest that some molecular mechanisms of retinotectal/collicular mapping diverge between birds and mammals [21], [41], [42], [43]. Thus, the results obtained with collicular EphAs are not necessarily applicable to tectal EphA3.

Given its decreasing rostro-caudal gradient in the chicken tectum, we hypothesized that EphA3 could account for the second activity necessary for retinotectal mapping along the rostro-caudal axis. Through functional *in vitro* and *in vivo* experiments, we demonstrated that the tectal gradient of EphA3 ectodomain is necessary to map nasal RGC axons on tectal surface by promoting nasal RGC axon growth toward the caudal tectum and inhibiting branching rostrally to their appropriate termination zone. Furthermore, the promotion of axon growth by tectal EphA3 allows us to explain how the optic fibers invade the tectum. Therefore, opposite tectal gradients of EphA3 and ephrin-As counterbalance each other during retinotectal mapping.

## Results

### Developmental patterns of expression of tectal EphA3, and retinal ephrin-As and tyrosine phosphorylated-EphA4

**Figure 1:**
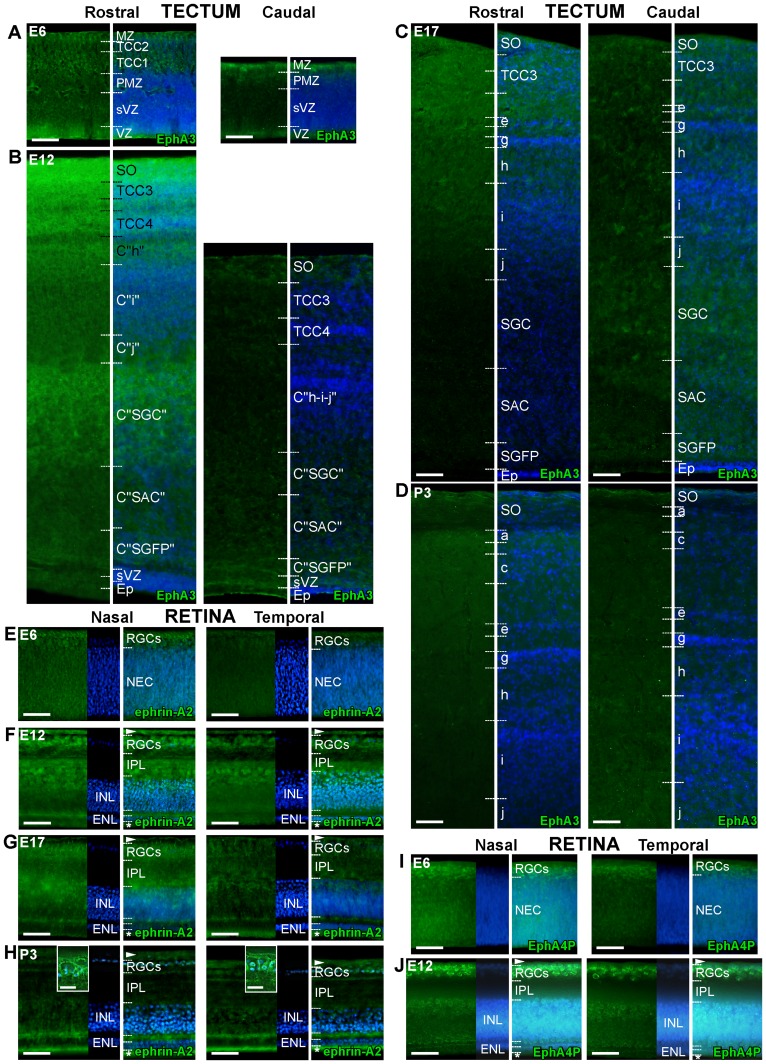
Developmental patterns of expression of tectal EphA3 and retinal ephrin-As and Tyr-602 phosphorylated-EphA4. (A–D) Immunofluorescence of EphA3 was performed in sections which extend along the rostro-caudal axis of tecta obtained from chicken embryos and early postnatal chicks. Photographs of rostral (left), and caudal (right) tectum obtained from the same section are successively shown at 6 (A), 12 (B), 17 days of development (C) (E6, E12, E17) and 3 postnatal days (P3) (D). Immunofluorescence (left) and merge image of immunofluorescence and nuclear labeling with Hoechst (right) of each area are shown. The lamination process is more advanced in rostral (left) than in caudal (right) tectum, so different developmental stages can be appreciated in different areas analyzed in the same age [3]. As lamination progresses, EphA3 is expressed along all the radial tectum in both areas, but the expression of superficial layers is higher in rostral than in caudal tectum. This pattern of expression is appreciated at E6 (A) and presents the highest level around E12 (B), decreasing postnatally (D). Ventricular zone (VZ), subventricular zone (SVZ), premigratory zone (PMZ), the transitory cell compartment 1 (TCC1), TCC2, TCC3, TCC4, C “stratum griseum periventriculare” (C “SGP”), C “stratum album centrale” (C “SAC”), C “stratum griseum centrale” (C “SGC”), C “j”, C “i”; C “h”; layers a, b, c, d, e, f, g, h, i, and j of the stratum griseum et fibrosum superficiale (SGFS); stratum opticum (SO) (for nomenclature see Material and Methods, [3], [5]). Scale bars  = 50 µm. (E-H). Immunofluorescence of ephrin-A2 (E–H) was performed in sections which extend along the naso-temporal axis of retinas obtained from E6 (E), E12 (F), E17 (G) and P3 (H). Photographs of nasal (left) and temporal (right) retinal areas obtained from the same section are successively shown. Immunofluorescence (left), nuclear labeling with Hoechst (middle) and merge image (right) of each area are shown. Ephrin-A2 is expressed in neuroepithelial cells and RGCs at E6 (E). When development progresses plexiform layers, amacrine cells and lastly external segments of photoreceptors are labeled (F–H). RGCs show ephrin-A2 in the soma, dendrites and the axon (insets). The expression of ephrin-A2 extends along all the retina, presenting a decreasing naso-temporal gradient mainly in the RGCs. This gradient presents the highest level of expression between E12 (F) and E17 (G), but persists postnatally when the expression of the photoreceptors increases (H). (I, J) Immunofluorescence of Tyr-602 phosphorylated-EphA4 was performed in sections which extend along the naso-temporal axis of retinas obtained when the gradient of ephrin-A2 is highly expressed (E6 and E12). Photographs of nasal (left) and temporal (right) retinal areas obtained from the same section are successively shown. Immunofluorescence (left), nuclear labeling with Hoechst (middle) and merge image (right) of each area are shown. Tyr-602 phosphorylated-EphA4 mainly expresses in RGCs and extends throughout all the retina, in a decreasing naso-temporal gradient at E6 (I) and E12 (J). Neuroepithelial cells (NEC), retinal ganglion cells (RGCs), optic fibers (arrowhead), photoreceptors (asterisk), external nuclear layer (ENL), inner nuclear layer (INL), inner plexiform layer (IPL). Scale bars  = 50 µm, inset: 10 µm.

To investigate the function of tectal EphA3 we first analyzed its developmental pattern of expression by performing immunohistochemistry during the embryonic development −4 to 18 days of incubation (E4 to E18)- and the early postnatal period –newly hatched, postnatal days 3 and 7 (P3 and P7)-. The study was achieved on sections coinciding with the rostro-caudal developmental gradient axis of the chicken optic tectum. This allowed us to distinguish between the most developed rostral pole (where the highest level of EphA3 is expressed), and the less developed caudal pole (where the lowest level of EphA3 is expressed) [3], [5], [18]. We showed that EphA3 is expressed along the main cellular layers during the embryonic development (Fig. 1A–D). The stratum opticum (SO) formed by the optic fibers is also labeled mainly in the rostral tectum where the EphA3-positive temporal fibers arrive. The levels of expression of EphA3 in the superficial layers – which include the SO and the retino-recipient layers- produce a decreasing rostro-caudal gradient which extends along the entire tectal axis. This gradient can be appreciated at E5–E6 –when the EphA3-positive temporal RGC axons have not yet invaded the tectum- and presents the highest level of expression around E12 when the retinotectal mapping is taking place. The EphA3 expression decreases postnatally when the retinotectal mapping has finished.

As axonal ephrin-As are the potential receptors for tectal EphA3, we analyzed the developmental pattern of expression of ephrin-A2 by performing immunocytochemistry on retinal sections coinciding with the naso-temporal axis from E4 to P7. We found that ephrin-A2 is expressed along the main cellular layers during embryonic development. Thus, it is expressed in neuroepithelial cells and RGCs starting from E4. During developmental progression plexiform layers, amacrine cells and lastly external segments of photoreceptors are labeled (Fig. 1E–H). The expression extends along the whole retina, presenting a decreasing naso-temporal gradient mainly in the RGCs. This gradient is particularly evident between E12 and E17 when retinotectal mapping is taking place, but it persists postnatally.

As EphA4 presents an even distribution along the naso-temporal retinal axis and it has been shown that axonal ephrin-As activate axonal EphA4 [18], [25], [44], we investigated by immunocytochemistry whether there is a relationship between the degree of EphA4 tyrosine-phosphorylation and ephrin-A expression. We showed that Tyr-602-phosphorylated-EphA4 is mainly expressed in RGCs and extends throughout the whole retina, in a decreasing naso-temporal gradient (Fig. 1I, J).

In order to confirm that ephrin-As and phosphorylated-EphA4 are expressed in the RGC axons, we performed immunocytochemistry on nasal and temporal retinal explants obtained from E6-E7 (HH29–30) chicken embryos and cultured during 24 hours. These stages of development have been chosen due to the fact that these explants present the best axonal growth *in vitro* [45], [46] and coincide with the period in which RGC axons invade the tectum [1], [2].

**Figure 2:**
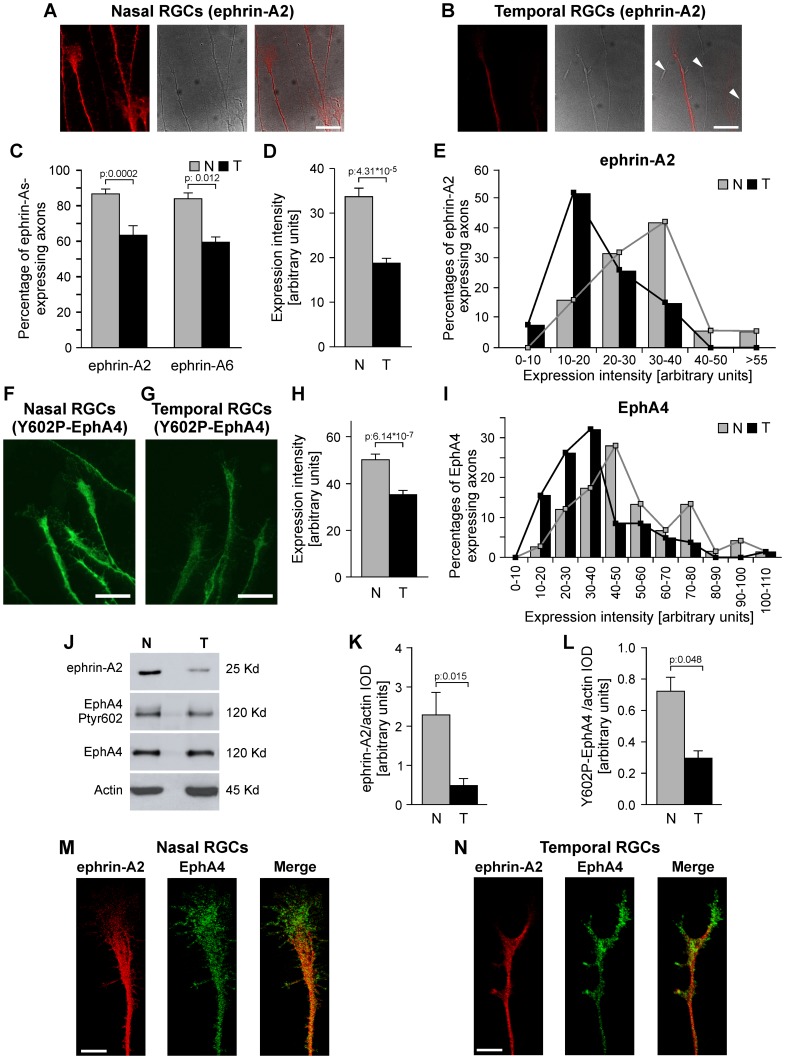
Ephrin-A2 and –A6 expression and EphA4 Tyr-602 phosphorylation decrease from nasal to temporal RGCs. (A–E) Expression gradients of ephrin-A2 and –A6 depend upon the level of expression of each RGC and the proportion of RGCs that express them. (A, B) Immunofluorescence against ephrin-A2 (left), phase-contrast (middle) and merge (right) of RGC axons grown during 24 hours from E7 nasal (A) and temporal (B) retinal explants are shown. Expression intensity and proportion of labeled axons are higher in nasal explants. Negative axons are depicted (arrowheads) in temporal explants. Scale bars  = 10 µm. (C) Quantification shows that a higher proportion of nasal axons express ephrin-A2 and –A6 (Student's t test, ephrin-A2: 4 independent experiments, 557 nasal and 733 temporal axons; ephrin-A6: 2 independent experiments, 326 nasal and 181 temporal axons). (D) Quantification of fluorescent intensity measured as integral optic density (IOD) in axons of nasal and temporal explants immunolabeled against ephrin-A2 shows that nasal axons present higher levels of expression (Mann Whitney test, 3 independent experiments, n: 52 nasal and 49 temporal axons). (E) Histogram of fluorescent intensity of one experiment shows that nasal and temporal axons form two partially overlapping populations where the major proportion of nasal axons presents higher levels of intensity than the major proportion of temporal axons. Similar results were obtained in the other experiments. (F, G) Immunofluorescence against Tyr-602 phosphorylated-EphA4 in nasal (F) and temporal (G) retinal explants are shown. Expression is higher in nasal axons. Scale bars  = 10 µm. (H) IOD is significantly higher in nasal axons (Mann Whitney test, 3 independent experiments, n: 75 nasal and 84 temporal axons). I. Histogram of fluorescent intensity shows that nasal and temporal axons form two partially overlapping populations where the major proportion of nasal axons presents higher levels of intensity than the major proportion of temporal axons. Similar results were obtained in the other experiments. (J) Western blot analysis against ephrin-A2, Tyr-602-phosphorylated-EphA4 and EphA4 of crude membrane fractions obtained from nasal and temporal retinas at E8. (K, L) Quantification of ephrin-A2/actin (K) and Tyr-602-phosphorylated-EphA4/actin ratios (L) from 3 similar experiments shows that nasal retina presents significantly higher levels of ephrin-A2 and Tyr-602-phosphorylated-EphA4 (Student's t test). (M, N) Confocal microphotographs of nasal (M) and temporal (N) axons grown from retinal explants immunolabeled against ephrin-A2 (red) and EphA4 (green) are shown. Right images are the merges. They show that there are more patches of overlapping (yellow) in the nasal axonal shaft and growth cone than in the temporal ones. Scale bars  = 5 µm. (Pearson's correlation coefficient: 0.62+/−0.011 versus 0.55+/−0.008, p: 0.045; Manders overlap coefficient: 0.82+/−0.002 versus 0.75+/−0.001, p: 9.56^*^10^−6^, 2 independent experiments; n: nasal RGCs: 30 axons, temporal RGCs: 26). Results are shown as mean +/− SE,. Nasal RGC axons (N), temporal RGC axons (T).

These studies showed that RGC axons (shafts and growth cones) express ephrin-A2 and –A6 in both nasal and temporal retinal thirds but presenting a decreasing naso-temporal gradient (Fig. 2A, B). To investigate whether this gradient is due to the number of axons which express ephrin-As or to the level of expression of each axon, we quantified the proportion of axons which express ephrin-A2 and –A6 and the intensity of labeling of each axon (Fig. 2A–E). We showed that not only temporal RGC axons express lower levels of ephrin-As with respect to nasal axons, but also a lower proportion of temporal RGC axons express ephrin-As if compared to nasal axons. The distribution profile appreciated in the histogram of fluorescent intensity (Fig. 2E) showed that nasal and temporal axons form two partially overlapping populations, the former presenting higher levels of expression.

RGC axons also present Tyr-602-phosphorylated-EphA4 in both nasal and temporal retinal thirds but showing a decreasing naso-temporal gradient (Fig. 2F, I). However, we could not find any axon which do not present Tyr-602-phosphorylated-EphA4. Quantification of the intensity of axon labeling showed that the gradient of Tyr-602-phosphorylated-EphA4 only depends upon the different levels of tyrosine-phosphorylation in each RGC axon (Fig. 2H). The profile observed in the corresponding histogram showed that nasal and temporal axons form two partially overlapping populations where the former presents the higher levels of expression (Fig. 2I). This pattern of distribution mimics that of the ephrin-A2, suggesting that ephrin-As could be related to EphA4-tyrosine-phosphorylation.

To confirm these distribution patterns, crude membrane fractions obtained from E8 nasal and temporal retinas were studied by Western blot to detect ephrin-A2, Tyr-602-phosphorylated-EphA4 and EphA4. These experiments confirmed that the levels of ephrin-A2 and Tyr-602-phosphorylated-EphA4 are significantly higher in nasal than in temporal retina (Fig 2J–L).

Explants from nasal and temporal retina were double immunolabelled for ephrin-A2 and EphA4 and analyzed with confocal microscopy. This showed the existence of big axonal segments in which only ephrin-A2 or EphA4 were expressed, but also significant areas of coexpression (Fig. 2M, N). Nasal RGC axons presented higher levels of colocalization than temporal RGC axons (Pearson's correlation coefficient: nasal: 0.62+/−0.011 versus temporal: 0.55+/−0.008, p: 0.045; Manders overlap coefficient: nasal: 0.82+/−0.002 versus temporal: 0.75+/−0.001, p: 9.56^*^10^−6^).

These results show that nasal RGC axons present higher levels of ephrin-As, colocalization of ephrin-A2 and EphA4, and tyrosine-phosphorylated EphA4 than temporal RGC axons. Thus, the high level of colocalization of ephrin-As and phosphorylated-EphA4 in nasal axons suggests that axonal ephrin-As might activate axonal EphA4 by phosphorylation. Furthermore, the differential distribution of ephrin-As and activated EphA4 between nasal and temporal RGC axons could explain the different behavior that nasal and temporal RGC axons present during retinotectal mapping (Figs. 1,2).

### EphA3 ectodomain stimulates nasal RGC axon growth in vitro

To evaluate our hypothesis that EphA3 ectodomain might stimulate nasal RGC axon growth, we compared RGC axon growth from nasal and temporal RGCs exposed to EphA3 ectodomain fused to Fc versus Fc as control *in vitro*.

All cultures employed in this work were prepared from retinas obtained from E7 (HH30–31) chicken embryos. Similar results were obtained with E6 (HH28–29) embryos (data not shown). We cultured both retinal explants and dissociated retinal neurons.

Retinal explants were plated on clustered EphA3-Fc or Fc (control). The substrates were produced by coating coverslips with an anti-human Fc polyclonal antibody, laminin and EphA3-Fc or Fc successively [47]. As optic fibers grow over a gradient of EphA3 on the tectal surface, clustered EphA3-Fc was assayed at different concentrations in order to evaluate whether its effect is concentration-dependent.

**Figure 3:**
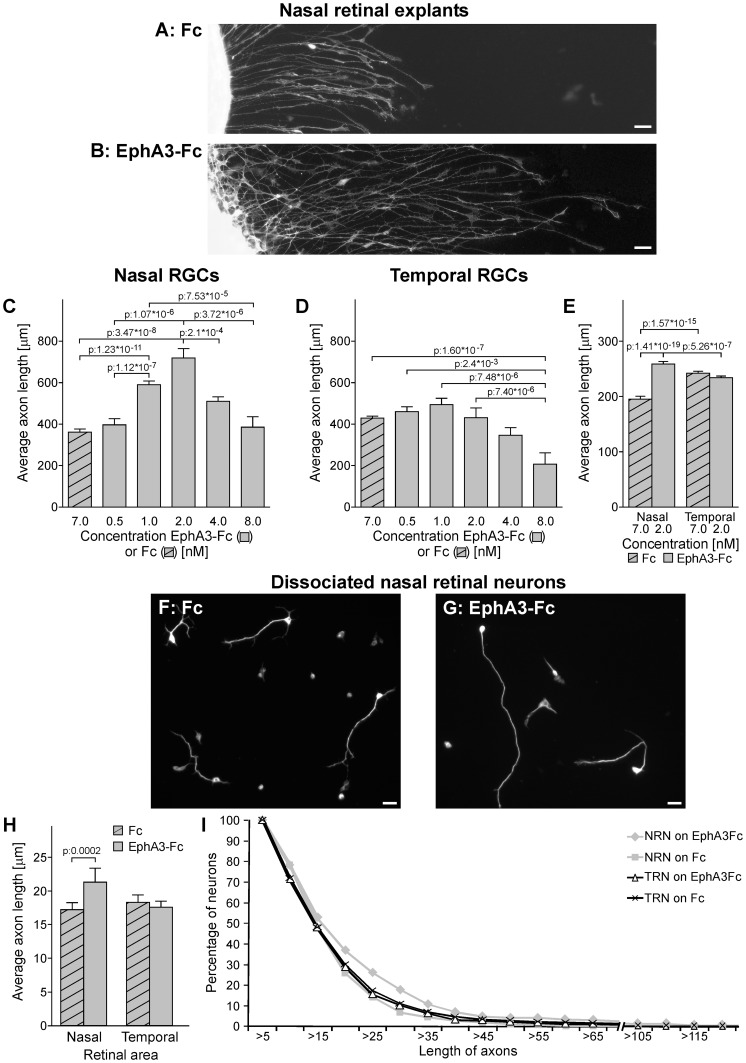
EphA3 ectodomain stimulates nasal RGC axon growth in vitro. (A, B) Microphotographs of nasal retinal explants grown on clustered Fc (A) or clustered EphA3-Fc at 2 nM (B). RGC axons grow longer on EphA3-Fc. Scale bars  = 20 µm. (C–D) Quantification of axon length of nasal (C) and temporal explants (D) grown on substrates formed by laminin and clustered Fc or EphA3-Fc at different concentrations. Axon length is indicated in µm and concentrations are indicated in nM of Fc or EphA3-Fc. Temporal explants grow longer axons than nasal ones in control conditions (p: 0.0002, compare first bar of nasal RGCs in C with first bar of temporal RGCs in D). EphA3-Fc increased nasal RGCs axon growth from 1 to 4 nM showing a peak at 2 nM. Temporal RGCs did not present any significant change in axon growth on EphA3-Fc between 0.5 and 4 nM and presented a significant decrease at 8 nM (ANOVA and Tukey postest, 3 independent experiments, n: 20 longer axons for explant, 3 explants for condition). (E) Quantification of axon length of nasal and temporal explants exposed to soluble clustered Fc or EphA3-Fc at 2 nM. Nasal RGC axons grow significantly longer with EphA3-Fc (ANOVA and Tukey postest, 3 independent experiments, n: 50 longer axons for explant, 3 explants for condition). (F–G) Dissociated nasal retinal neurons immunolabeled against neuron specific βIII tubulin. They present longer axons on clustered EphA3-Fc at 2 nM (G) than on clustered Fc (F). Scale bars  = 10 µm. (H) Quantification of axon length of nasal and temporal dissociated retinal neurons grown on clustered Fc or EphA3-Fc at 2 nM. Nasal retinal neurons grow significantly longer axons on EphA3-Fc. (ANOVA and Tukey postest, 3 independent experiments, n: nasal retinal neurons on EphA3-Fc: 288, nasal retinal neurons on Fc: 328, temporal retinal neurons on EphA3-Fc: 636, temporal retinal neurons on Fc: 646). (I) The plot depicts the distribution of axon length of nasal and temporal dissociated retinal neurons (NRN and TRN) grown on EphA3-Fc versus Fc. Values given on the y-axis indicate the proportion of retinal neurons which axons reach the length shown on the x-axis. Nasal retinal neurons present a higher proportion of axons between 20 and 40 µm (n: nasal retinal neurons on EphA3-Fc: 288, nasal retinal neurons on Fc: 328, temporal retinal neurons on EphA3-Fc: 636, temporal retinal neurons on Fc: 646). Results are shown as mean +/– SE.

EphA3-Fc induced a concentration-dependent response on axon growth which was specific for nasal and temporal RGCs (Fig. 3A–D). Overall, nasal RGCs presented shorter axons than temporal RGCs in control conditions. The former were 18.95 %+/−4.8 % shorter than temporal axons, (compare first bar of Fig. 3C with first bar of Fig. 3D). EphA3-Fc significantly increased nasal RGC axon growth between 1 nM and 4 nM (Fig. 3C), presenting the maximal effect at 2 nM (this concentration increased axon length in 128.14 %+/−12.17 % with respect to control). These concentrations of EphA3-Fc did not produce any significant effect on temporal RGC axon growth (Fig. 3D). However, higher concentrations of EphA3-Fc (8 nM) did not present any significant effect on nasal RGC axon growth (Fig. 3C) whereas decreased temporal RGC axon growth (Fig. 3D).

In order to exclude that EphA3-Fc-stimulated axon growth could be only the consequence of adhesive effects produced by the studied molecule, we exposed nasal and temporal explants -grown on poly-L-lysine and laminin- to soluble clustered EphA3-Fc at 2 nM (concentration which produced the maximal effect on nasal RGC axon growth) or soluble clustered Fc (Fig. 3E). We showed that temporal RGCs grew longer axons than nasal ones in control conditions and that soluble clustered EphA3-Fc significantly stimulated nasal RGC axon growth without producing any significant effect on temporal RGCs. This means that adhesive interactions between axonal ephrin-As and EphA3 ectodomain are not completely necessary to explain the effect of EphA3 ectodomain on axon growth.

To evaluate the effect of EphA3 ectodomain on individual RGCs which do not present interaxonal contacts, nasal and temporal dissociated retinal neurons were cultured on anti-human Fc polyclonal antibody, laminin and Fc or EphA3-Fc at 2 nM (Fig. 3F, G). Neurons were recognized throughout their morphology and by immunolabeling against neuron specific βIII tubulin. Only the longest neurite of each neuron exceeding twice the cell body diameter was considered as a RGC axon and taken into account for quantification. Nasal retinal neurons presented longer axons on EphA3-Fc than on Fc whereas temporal ones did not present any significant difference in the two conditions (Fig. 3H, I). These results cannot exclude a role of interaxonal contacts in the effect of EphA3 ectodomain on RGC axon growth but this suggests that axonal interaction is not completely necessary for this effect.

**Figure 4:**
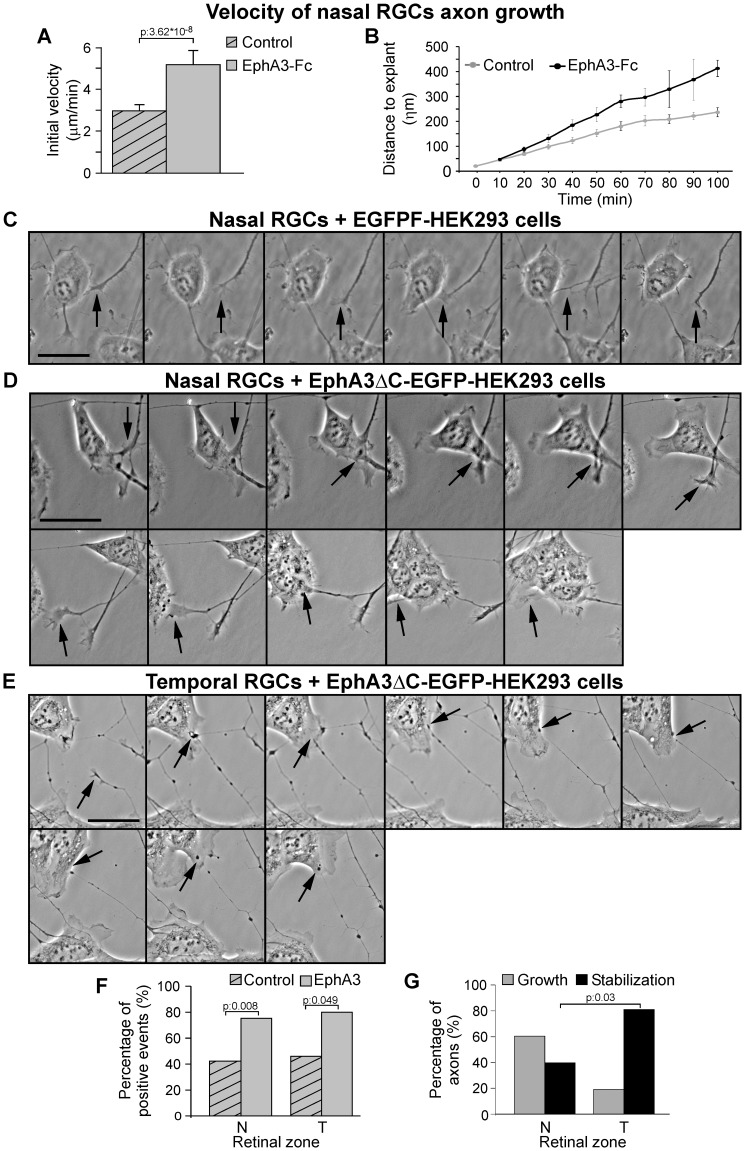
EphA3 ectodomain increases the velocity of nasal RGCs axon growth and RGC axons choose growing on EphA3-expressing cells. (A) Quantification of axonal growth rate (during the first 100 minutes since axon appearance) of nasal explants exposed to soluble clustered EphA3-Fc or Fc (Student's t test, 3 experiments, n: 12 axons for explant, 3 explants for condition). Results are shown as mean +/− SE. (B) Quantification of distance covered by nasal axons exposed to soluble clustered EphA3-Fc and Fc during the first 100 minutes after their appearance. Nasal axons grow faster with EphA3-Fc (p: 3.62^*^10^−8^, Student's t test). (C–E) Representative microphotographs of a time-lapse experiment. They show the behavior of axonal growth cones of nasal and temporal explants making contact with EphA3ΔC-EGFP-transfected-HEK293 cells or EGFP-F-transfected-HEK293 cells (control) (see arrows). (C) Nasal growth cones indistinctly attach or retract from EGFP-F-transfected-293 cells (Total time: 147 minutes). Similar results were obtained with temporal growth cones. (D) Nasal axons tend to grow along EphA3ΔC-EGFP-transfected-293 cells (Total time: 183 minutes) whereas (E) temporal axons tend to adhere to EphA3ΔC-EGFP-transfected-293 cells (Total time: 471 minutes). Scale bars  = 20 µm (see Videos S1, S2, S3). (F) Proportions of positive events (elongation or adhesion) produced by growth cones making contact with EphA3ΔC-EGFP-transfected-HEK293 cells versus EGFP-F transfected-HEK293 cells. Positive events significantly increase with EphA3 expressing-cells (Fisher's exact test; n: 79 nasal growth cones, 39 temporal growth cones; 3 nasal and 3 temporal explants for condition). (G) Discrimination of positive events between elongation and adhesion produced by growth cones making contact with EphA3ΔC-EGFP-transfected-HEK293 cells. Nasal axons present a significantly higher proportion of elongation than temporal ones (Fisher's exact test; n: 58 growth cones; 3 nasal and 3 temporal explants). Nasal (N), temporal (T).

To investigate the dynamics of axon growth in retinal explants, we performed time-lapse experiments. These assays revealed that nasal RGC axons started to grow after 9 hours of exposure to both the soluble clustered EphA3-Fc or Fc control, but axons grew faster when exposed to EphA3-Fc than to Fc (Fig. 4A, B). Hence, EphA3-Fc enhances nasal axon growth without modifying the time point of initial axon formation.

### RGC axons choose growing on EphA3 ectodomain-expressing cells

To determine whether RGC axons choose growing on EphA3 ectodomain expressed on cell surfaces, such as the case in the rostral optic tectum, we performed time-lapse experiments. We made cocultures of nasal or temporal retinal explants and HEK293 cells stably-transfected with the extracellular domain of EphA3 (EphA3ΔC–EGFP) or the membrane-targeted farnesylated EGFP-F. We examined the behavior of growth cones making contact with the transfected HEK293 cells. Positive responses included adhesion to and elongation over the target cells, whereas negative responses included collapse and withdraw from the cells. These experiments showed that both nasal and temporal growth cones did not present any significant difference between positive and negative events when they made contact with EGFP-F-expressing cells. However, both nasal and temporal growth cones showed a significantly higher proportion of positive responses when they made contact with EphA3-expressing cells (Fig. 4C–F; see Videos S1, S2, S3). The majority of nasal axons elongated over EphA3-expressing HEK293 cells, whereas the majority of temporal axons adhered but only few extended over them (Fig. 4G). These results are consistent with the *in vivo* phenomenology, where nasal axons extend over the EphA3-expressing cells toward the caudal tectum and temporal axons navigate for a shorter distance before establishing their connections with the EphA3-expressing cells. Furthermore, these results agree with the previous *in vitro* experiments in which EphA3-Fc increased nasal RGC axon growth but did not modify or decreased temporal RGC axon growth depending on its concentration (Fig. 3C, D).

Taken together, time-lapse experiments suggest that the EphA3 ectodomain behaves as a guidance cue, which is chosen to grow on by the RGC axons and may stimulate nasal RGC axon growth toward the caudal tectum.

### EphA3 ectodomain decreases the density of axonal filopodia in vitro

**Figure 5:**
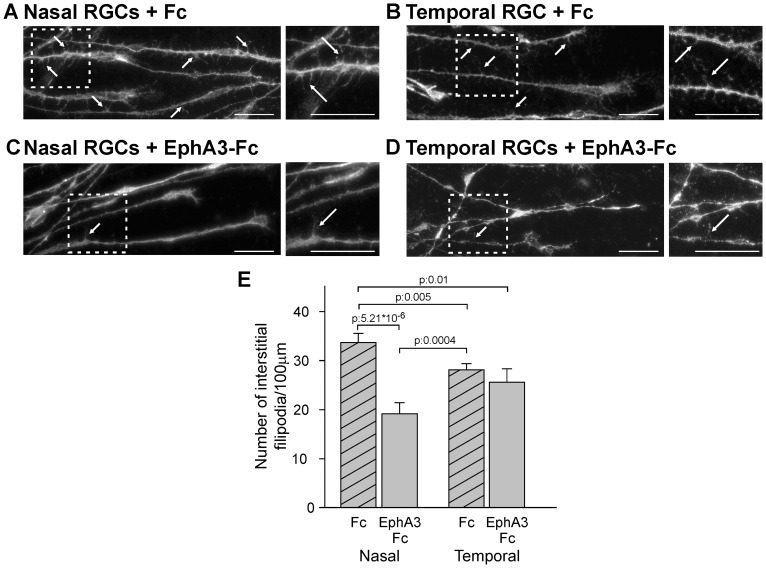
EphA3 ectodomain decreases the density of interstitial filopodia in nasal RGC axons. A–D. Representative microphotographs of axons grown from nasal (A, C) and temporal (B, D) retinal explants exposed to soluble clustered Fc (A, B) or EphA3-Fc (C, D). Axons are labeled with Alexa 488-phalloidin. Arrows depict representative interstitial filopodia. Insets show filopodia at higher magnification. Scale bars  = 20 µm. (E) Quantification of filopodia number/100 µm of axon shafts. Nasal axons present higher density of interstitial filopodia and EphA3-Fc significantly decreases the density of interstitial filopodia in nasal RGC (ANOVA and Tukey postest, 3 experiments, n: 8 axons for explant, 4 explants for condition). Results are shown as mean +/− SE.

As the topographic-specific formation of interstitial branches is considered a critical event in chicken and mice retinotopic mapping [2], [6], we investigated whether the EphA3 ectodomain regulates the density of axonal interstitial filopodia -the precursors of axonal branches [48], [49]- by performing an *in vitro* assay [37], [39], [49]. We exposed axons of nasal and temporal retinal explants to soluble clustered Fc or EphA3-Fc (2 nM) for 2 hours and quantified the axonal filopodia observed in phase-contrast or stained with Alexa 488-conjugated phalloidin after fixation. Axons with a similar low degree of interaxonal contacts were selected. We showed that nasal axons presented 17.49 %+/−6.58 % higher density of interstitial filopodia than the temporal ones and that EphA3-Fc significantly decreased the density of interstitial filopodia by 43.2%+/−5.6% in nasal RGC axons (Fig. 5A–E). This demonstrates that EphA3 ectodomain also reduces the density of axonal filopodia of the nasal RGCs *in vitro*.

### Misexpression of EphA3 ectodomain in the optic tectum disrupts retinotectal mapping by increasing axon growth and inhibiting axon branching of nasal RGCs

Our *in vitro* results suggest that EphA3 expressed in a decreasing rostro-caudal gradient along the chicken optic tectum might stimulate the growth of nasal RGC axons toward the caudal tectum and inhibit branching rostrally to the appropriate termination zone. To corroborate this hypothesis, we employed an RCAS retroviral vector to overexpress in the tectum the EphA3 ectodomain fused to EGFP replacing the cytoplasmic domain (EphA3ΔC-EGFP) or to express the membrane-targeted farnesylated EGFP-F as control. Injections of RCAS were made into the tectum at E2-E2.5 (HH13–16). To investigate the topographic mapping, small focal injections of DiI diluted in ethanol (10%) were made into peripheral areas of the temporal or nasal retina between E9 (HH35) and E16 (HH43). Embryos were dissected between E11 (HH37) and E19 (HH45). The location of labeled RGC bodies was determined in whole mounts of each retina (see Fig S1A) and the location of optic fibers and regions of EphA3 ectodomain overexpression were determined in whole mounts of the contralateral optic tectum. Optic fibers were observed as long axons in the more superficial focal plane of the tectal whole mount because they form the SO extending along the rostro-caudal axis of the tectum. Domains of EGFP expression were recognized as dense green fluorescent areas in which different types of cells and processes could be appreciated at different focal levels along the thickness of the tectal whole mount.

We also used vibratome sections to analyze the radial location and morphology of EphA3ΔC-EGFP or EGFPF-expressing cells. At every examined developmental stage, cells expressing EphA3ΔC-EGFP or EGFPF formed columns that extended throughout the entire radial thickness of the tectum, including cellular processes located in the SO and making contact with the optic fibers (see Fig. S1B, C in the supporting information). Expression of EphA3ΔC-EGFP did not affect the size, morphology, developmental gradient, or laminar organization of the tectum in comparison with EGFPF expression.

We performed two types of analyses to define whether EphA3 ectodomain misexpression alters topographic mapping: 1) analysis of the behavior of nasal and temporal optic fibers which make contact with EphA3 overexpressing-tectal areas versus EGFPF expressing-tectal areas and 2) topographic analysis performed by relating the location of labeled-RGC somas along the naso-temporal axis with the location of their optic fibers along the tectal rostro-caudal axis. For this purpose, both retinal and tectal whole mounts were divided into three areas (nasal, central and temporal retina, and rostral, intermediate and caudal tectum). In both types of analysis, the proportions of optic fibers which passed through and formed terminations zones were compared between EphA3 overexpressing and control tecta. Labeled superficial fibers which passed along the total analyzed area were considered passing optic fibers when presenting neither increased density of filopodia nor ramified branches. Labeled superficial fibers which presented an arborization located at the end or closed to the end of the axon over the analyzed area were considered fibers forming termination zones [6], [25].

**Figure 6:**
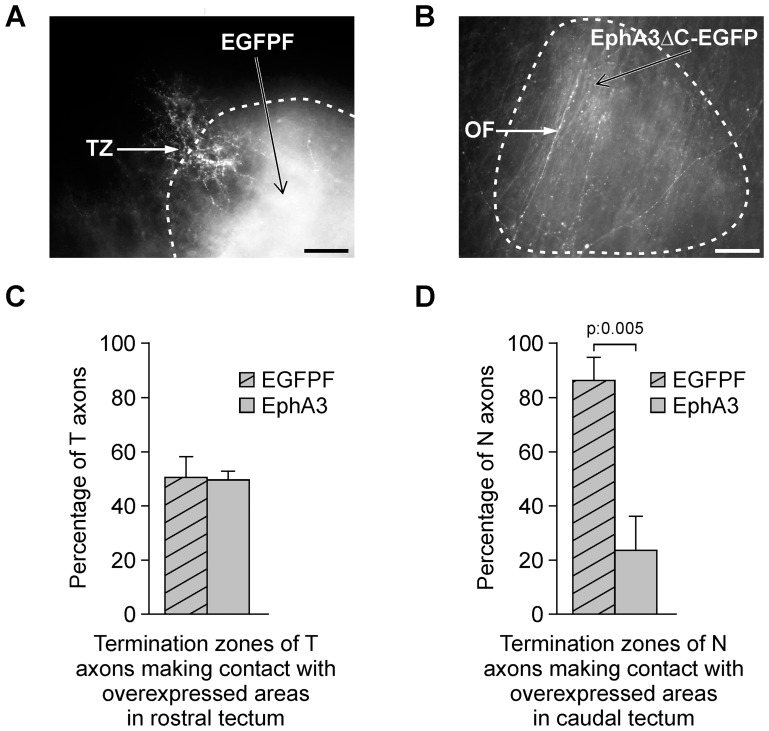
EphA3 ectodomain overexpression stimulates nasal optic fibers (OFs) passing throughout and inhibits termination zones (TZs) formation. After infection of the optic tectum at E2 with RCAS-BP-B-EGFPF (control) (A) or with RCAS-BP-B-EphA3ΔC-EGFPN3 (B) and DiI labeling of the naso-dorsal retina at E16 (HH42), the tectum was analyzed in whole mounts at E18 (HH44). (A, B) Microphotographs show a termination zone (TZ) formed by nasal optic fibers in an EGFPF-positive domain located in the caudal tectum (A) and nasal optic fibers (OFs) passing throughout an EphA3ΔC-EGFP- positive domain located in the caudal tectum (B). Dotted lines demarcate the overexpressed regions. Scale bars  = 50 µm. (C, D) Comparison between the proportions of temporal (C) and (D) nasal RGC axons (T RGC and N RGC) which form TZs in the areas which express EGFPF versus EphA3ΔC-EGFP. Temporal RGC axons were evaluated in the rostral tectum whereas nasal RGC axons were evaluated in the caudal tectum. (C) No significant difference is detected between the proportion of temporal RGC axons which form TZs in EphA3ΔC-EGFP-positive regions and in EGFPF-positive regions (control) in rostral tectum. (D) A significantly lower proportion of nasal axons form TZs in EphA3ΔC-EGFP-positive regions than in EGFPF-positive regions (control) in caudal tectum (Student's t test, n: 4 EphA3ΔC-EGFP-overexpressed tecta versus 7 control tecta for temporal RGCs; n: 5 EphA3ΔC-EGFP-overexpressed tecta versus 4 control tecta for nasal RGCs). Results are shown as mean +/− SE.

To analyze the effects on the behavior of retinal optic fibers exerced by the overexpression of EphA3 ectodomain on the tectum, we compared the proportions of nasal and temporal axons which passed throughout or formed termination zones in contact with EphA3ΔC-EGFP versus EGFPF positive tectal regions. RGC axons were evaluated in the corresponding target area. Results were analyzed at the time point in which a mature retinotectal projection was established (between E15–HH41– and E19–HH45-) [6]. We found that a significantly higher proportion of nasal optic fibers passed through EphA3ΔC-EGFP-positive areas (75.94 %+/−12) in comparison with EGFPF-positive areas in the caudal tectum (13.33 %+/−8.16,). Conversely, a significantly lower proportion of axons formed termination zones in EphA3ΔC-EGFP-positive areas (Fig. 6A, B, D). However, temporal RGC axons did not show any significant difference in terms of proportions of axons which produce termination zones after making contact with EphA3ΔC-EGFP-positive areas (49.29%+/−3.52%) versus EGFPF-positive areas (50.68%+/−7.58%) in the rostral tectum (Fig. 6A, C). These results show that the EphA3 ectodomain stimulates axon growth and inhibits termination zones formation on nasal RGCs *in vivo* whereas it does not present any significant effect on temporal RGCs.

**Figure 7:**
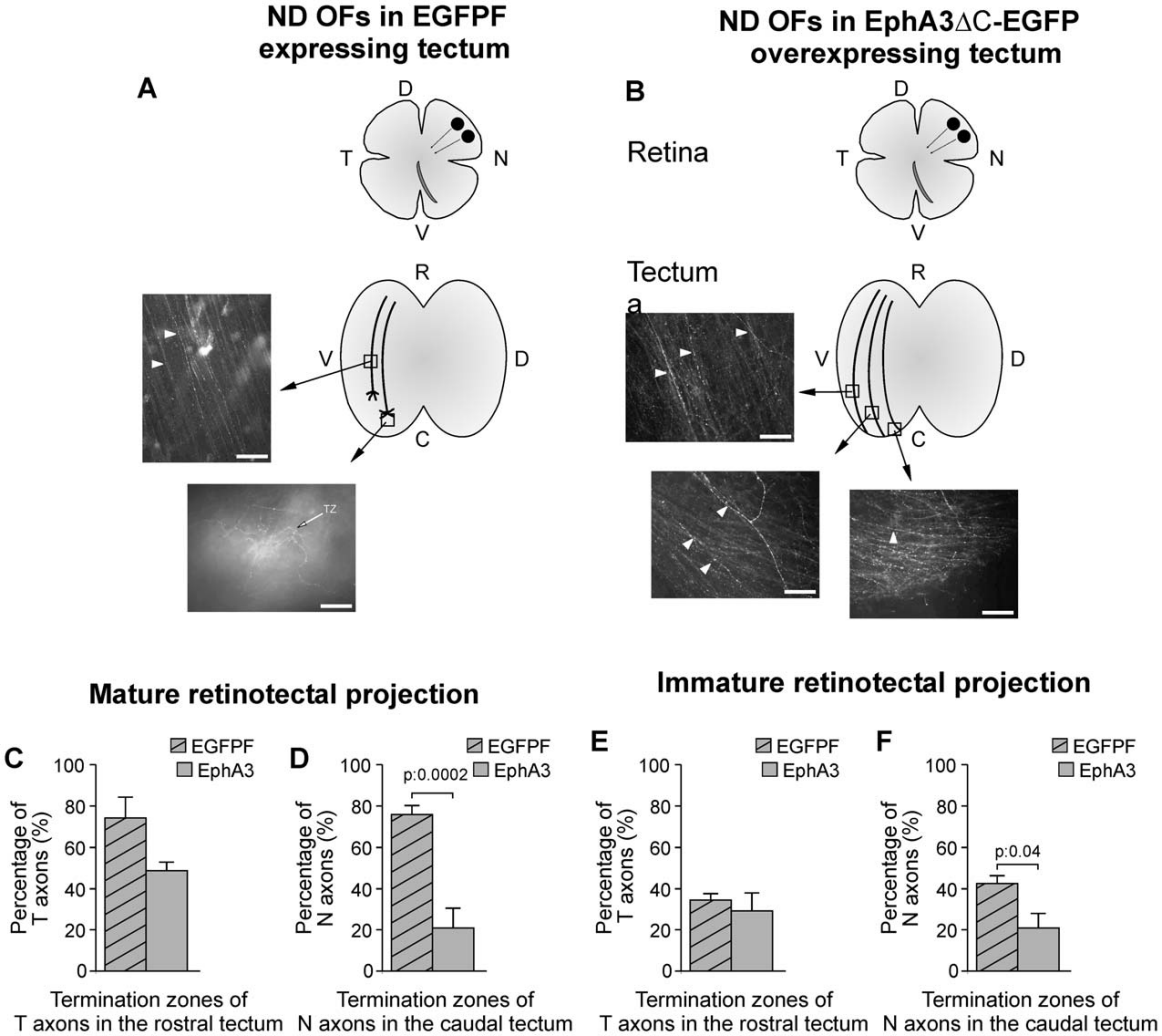
EphA3 ectodomain overexpression alters the topographic map formation. After infection of the optic tectum at E2 with RCAS-BP-B-EGFPF (control) (A) or with RCAS-BP-B-EphA3ΔC-EGFPN3 (B) and DiI labeling of the naso-dorsal retina at E16, the retina and the tectum were analyzed in whole mounts at E18 (A, B). Graphs represent retinal whole mounts labeled in naso-dorsal areas (ND) with DiI (black circles) (upper) and tectal whole mounts where the corresponding DiI labeled RGC axons (drawings) are shown in microphotographs. (A) Optic fibers (OFs) pass throughout the intermediate tectum (arrowheads) and form a termination zone (TZ) (arrow) over an area of EGFPF expression in the corresponding target area (caudal tectum) in a control tectum. (B) OFs pass through the target area (caudal tectum) without producing TZs (arrowheads) in a tectum where EphA3ΔC-EGFP was overexpressed. N: nasal, T: temporal, V: ventral, D: dorsal, R: rostral, C: caudal. Scale bars  = 50 µm. (C–F) Graphs show the proportions of temporal (C, E) and nasal (D, F) OFs which form TZs in the rostral and the caudal tectum respectively in EGFPF-expressing tecta versus EphA3ΔC-EGFP-overexpressed tecta. (C, D) represent the proportions of TZs observed after the remodeling period -E15 (HH41)-E19 (HH45)- whereas (E, F) represent the proportions of TZs observed during the remodeling period -E11 (HH37)-E14 (HH40)-. (D) Nasal RGC axons (N RGC) present a significantly lower proportion of TZs in the caudal area of EphA3ΔC-EGFP-overexpressed tecta between E15 and E19 whereas (C) temporal RGC axons (T RGC) do not present any significant difference in the rostral area of EphA3ΔC-EGFP-overexpressed tecta between E15 and E19 (Student t test, n: 6 EphA3ΔC-EGFP-overexpressed tecta versus 7 control tecta for nasal RGC; n: 4 EphA3ΔC-EGFP-overexpressed tecta versus 10 control tecta for temporal RGCs). (F) Nasal RGCs present a significantly lower proportion of TZs in the caudal area of EphA3ΔC-EGFP-overexpressed tecta between E11 and E14 whereas (E) temporal RGCs do not present any significant difference between the rostral areas of the EphA3ΔC-EGFP-overexpressed tecta and control tecta (Student t test; n: 6 EphA3ΔC-EGFP-overexpressed tecta versus 41 control tecta for nasal RGCs and 5 EphA3ΔC-EGFP-overexpressed tecta versus 53 control tecta for temporal RGCs). N: nasal, T: temporal, V: ventral, D: dorsal, R: rostral, C: caudal. Results are shown as mean +/− SE.

To analyze the retinotectal mapping along the rostro-caudal axis, we determined the proportions of temporal and nasal RGC axons which passed through or formed termination zones in the corresponding target area along the tectal rostro-caudal axis when a mature retinotectal projection was established (between E15 and E19). This analysis showed that EphA3 ectodomain overexpression significantly decreased the proportion of termination zones formed by nasal RGC axons in the caudal tectum (21.18%+/−9.47) when compared to the control-EGFPF-expressing tecta, (75.82%+/−4.84) (Fig. 7A, B, D). Thus, EphA3 ectodomain overexpression increased the proportion of nasal optic fibers which passed through the corresponding target areas and projected to the caudal end of the tectum. However, EphA3 ectodomain overexpression did not produce any significant change in the proportion of termination zones formed by temporal RGC axons in the rostral tectum (74.38%+/−9.89% in EGFPF-expressing tecta versus 49.29%+/−3.52% in EphA3ΔC-EGFP overexpressing tecta) (Fig. 7C). The penetrance of this phenotype was 100%. These results demonstrate that EphA3 ectodomain overexpression alters topographic map formation by stimulating nasal axon growth to the caudal tectum and decreasing branch formation.

During the remodeling period (between E11-HH37- and E14 –HH40-), EphA3 ectodomain overexpression produced a significant decrease in the proportion of termination zones formed by nasal RGC axons in the caudal tectum (20.70%+/−7.28) with respect to EGFPF-expressing tecta (42.47%+/−3.8) (Fig. 7.F). There was not any significant change in the proportion of termination zones formed by temporal RGC axons in the rostral area of EphA3-overexpressing-tecta (29.15%+/−8.52) with respect to EGFPF-expressing tecta (34.28%+/−3.13) (Fig. 7E). Differences observed between EphA3-overexpressing-tecta and control tecta were significantly lower during the remodeling period than in the postremodeling period. This is due to the fact that in control conditions several immature axons pass throughout their target areas before forming termination zones and pruning the leading axonal segment during the remodeling period [6]. However, the significant disruption in the location of nasal RGC axons appreciated during the remodeling period shows that tectal EphA3 is involved in topographic map formation from early stages.

Taken together, our results show that tectal EphA3 is involved in retinotectal mapping, stimulates nasal RGC axon growth and inhibits nasal axon branching *in vitro* and *in vivo*.

## Discussion

This work aims at investigating the nature of the second gradient of activity which counterbalances the repulsive effect of tectal ephrin-As for mapping RGC axons along the rostro-caudal tectal axis.

We demonstrated that EphA3 stimulates nasal RGCs axon growth and decreases the density of interstitial filopodia of the nasal RGC axons *in vitro.* We also showed that tectal overexpression of EphA3 ectodomain disrupts the retinotectal mapping sending nasal RGC axons toward the caudal tectum and preventing them from producing termination zones in their appropriate target area. Both types of experiments strongly suggest that the tectal EphA3 is involved in the establishment of topographic ordered retinotectal connections by stimulating nasal axons growth toward the caudal tectum and preventing them from branching rostrally to their appropriate target area. Therefore, this molecule behaves as a membrane-bound ligand and becomes, at least partially, responsible for the proposed second force necessary to explain the retinotectal mapping along the rostro-caudal axis.

### Tectal EphA3 stimulates nasal RGC axon growth toward the caudal tectum and inhibits branching rostrally to their appropriate target area

*In vitro* experiments demonstrated that EphA3 ectodomain produces a concentration dependent effect on RGC axon growth suggesting that it may act throughout a gradient distribution and this effect is specific to the RGC location (Fig. 3). It was also shown by time-lapse experiments that nasal and temporal RGC axons choose growing on cellular expressed-EphA3 ectodomain. Nasal RGC axons grow over EphA3 expressing-HEK293 cells whereas temporal RGC axons tend to adhere and grow slowly over these cells (Fig. 4C–G, see videos S1, S2, S3). These time-lapse results are compatible with the behavior of optic fibers *in vivo*, where nasal RGC axons grow over the rostral tectum –in which the EphA3 is highly expressed- and temporal RGC axons navigate a shorter distance before establishing their termination zones.

Moreover, our *in vitro* results showed that EphA3 ectodomain reduces the number of interstitial filopodia in nasal axon shafts (Fig. 5) suggesting that tectal EphA3 might inhibit nasal optic fibers branching rostrally to their appropriate target. Consequently, misexpression of EphA3 ectodomain stimulates nasal RGC axon growth toward the caudal tectum and inhibits axon branching (Figs. 6B, D and 7B, D).

Taken together, our *in vitro* and *in vivo* results support the idea that the tectal gradient of EphA3 is required for retinotectal mapping by stimulating nasal RGCs axon growth toward the caudal tectum and inhibiting termination zones formation rostrally to their appropriate target area.

The role of Eph-ephrin system was not studied regarding specific populations of RGC axons which grow at different stages of development. The mapping disruption produced by EphA3 overexpression from early stages of development (Fig. 7E, F) suggests that tectal EphA3 is necessary for guidance of pioneer axons. Furthermore, our finding that EphA3-Fc stimulates nasal axon growth in dissociated cultures of retinal neurons suggests that axonal interactions are not completely necessary to explain the effect of EphA3 ectodomain on axon growth. On the other hand, given that the gradients of tectal EphA3 and axonal ephrin-As and tyrosine-phosphorylated-EphA4 are highly expressed along all the process of retinotectal mapping (Fig. 1), it is suggested that EphA-ephrin-A system is involved in axon guidance not only of pioneer axons but of the trailing ones as well. This is consistent with the hypothesis that the level of axonal fasciculation and segregation can be influenced by Eph-ephrin system. Accordingly, it was suggested that axons expressing high levels of EphAs could be segregated from those which express high levels of ephrin-As by a forward interaxonal signaling [50]. Finally, it was postulated that EphA-ephrin-As fiber/target interactions play a main role in the global mapping whereas EphAs-ephrin-As fiber/fiber interactions are involved in local distribution of axons in later stages of development [8], [13], [35]. However, the relative role of EphAs-ephrin-As in target/fiber and fiber/fiber interactions is an issue that remains unresolved.

The developmental patterns of expression of axonal ephrin-As and EphAs, the level of their colocalization and the coincident distribution of ephrin-As and the tyrosine-phosphorylated-EphA4 suggest that ephrin-As could activate EphA4 by tyrosine-phosphorylation. Furthermore, the differential distribution of ephrin-As and activated-EphA4 between nasal and temporal RGC axons could explain the different response that nasal and temporal RGC axons present when they are exposed to EphA3 during retinotectal mapping. These results suggest the existence of two possible molecular mechanisms of action for tectal EphA3 on RGC axons. Thus, EphA3 could act throughout ephrin-As reverse signaling as it was supported in mice [36], [37], [38], [39] and/or throughout indirect regulation of axonal EphAs forward signaling as it was suggested in chicks [25], [33], [34], [44], [51].

### Other models proposed about the second force which participates in the retinotectal/collicular mapping along the rostro-caudal axis

EphA7 and EphA8 [24], [52] have been also postulated as responsible for the second gradient that regulates mapping along the rostro-caudal axis of the retinotectal/collicular system. Thus, rostrally shifted ectopic termination zones of nasal RGCs were obtained in EphA7 knock-out mice [24] and caudally shifted nasal RGC axons were obtained by overexpressing EphA8 [52]. These results are consistent with the caudally shifted nasal RGC axons obtained by overexpressing the tectal EphA3 (Fig. 7). Nevertheless, as EphA7-Fc repelled retinal axons in stripe assays, a repellent instead of a stimulating effect on axon growth was attributed to EphA7. In our experimental conditions, however, EphA3 ectodomain was chosen by the RGC axons and stimulated nasal RGC axon growth. With respect to this apparent discrepancy, it should be considered that EphA7-Fc was used in stripe assays [24], [36], [37], [38], [39] at 30 fold higher concentrations than the highest concentration of EphA3-Fc used here (Fig. 3C, D). At similar concentrations to the higher ones used in our experiments, EphA7 did not affect nasal RGC axon growth, but -in agreement with our results- decreased the density of interstitial filopodia [37]. Thus, the different responses of RGC axons to EphA7 and EphA3 ectodomains may represent the effects of different concentrations of EphAs *in vitro*.

**Figure 8:**
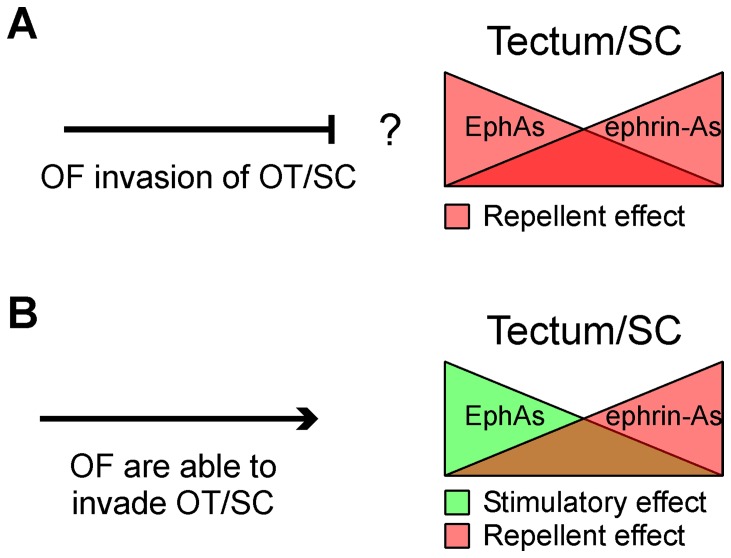
General models to account for topographic mapping along the rostro-caudal axis of the retinotectal/collicular system. In both models (A and B) opposing gradients of ephrin-As and EphAs establish the local addresses in the tectum/colliculus whereas opposing gradients of ephrin-As and EphAs establish the relative sensitivity of axons according to the RGC bodies location. Tectal/collicular ephrin-As inhibit temporal RGCs axon growth and termination zone formation. (A) In this model collicular EphA7 or EphA8 repel nasal RGC axon growth and inhibit termination zone formation. Repellent effect of EphAs on axon growth would prevent RGC axons from invading the colliculus. (B) In our model tectal EphA3 stimulates nasal RGCs axon growth and inhibits termination zone formation. This allows us to explain how RGC axons invade the tectum. Optic fibers (OF), Optic tectum (OT), superior colliculus (SC).

The question about the effect of the second tectal/collicular gradient on RGC axon growth (stimulation versus repulsion) implies fundamental consequences on the way the optic fibers invade the tectum/colliculus. As optic fibers invade the tectum/colliculus throughout the area where the highest concentration of EphAs are expressed, the repellent effect of EphA7 would prevent optic fibers from invading the target (Fig. 8A). However, the existence of a molecular gradient of EphA3, which stimulates axon growth throughout it, can explain how the optic fibers invade the tectum/colliculus [1] (Fig. 8B).

On the another hand, the works about mice EphA7 [24], [36], [37], [38], [39] and our work about chicken EphA3 agree because both EphAs diminish the density of interstitial filopodia in nasal RGC axons *in vitro* and inhibit branching rostrally to the appropriate target area *in vivo*. It was shown that BDNF induces axon branching by promoting the formation of a TrkB/ephrin-A5 complex and that EphA7-Fc inhibits this effect throughout p75 neurotrophin receptor/ephrin-A5 complex activation [37]. These data suggest that BDNF plays a general role of branching stimulation and the caudal ephrin-As and the rostral EphAs repress -in a topographic specific way- branching caudally or rostrally to the appropriate termination zone [2].

This model does not discard the role of other cues in mapping along the rostro-caudal axis of retinotectal system, such as engrailed [53], [54] and repulsive guidance molecule [55].

Our results are very useful because neural topographic ordered connections are the base of the nervous system normal function and their restitution is the base of any therapeutic approach.

## Materials and Methods

### Animals

Pathogen free fertilized White Leghorn chicken (Gallus gallus) eggs were obtained from Rosenbusch Institute (Buenos Aires). They were incubated at 38°C and 60% relative humidity. Developmental stages were recorded according to Hamburger and Hamilton Table of Stages (HH) [56]. After hatching animals were reared under standard housing conditions and supplied with food and water ad libitum. Animals were treated following the Guide for the Care and Use of Laboratory Animals from the Institute of Laboratory Animals Resources, Commission of Life Sciences, National Research Council, USA, and approved by the Council for Care and Use of Experimental Animals from University of Buenos Aires. Embryos were removed from the eggs, decapitated and dissected out in ice-cold Hank's Balanced Salt Solution (HBSS) or in 0.1 M sodium phosphate buffer (pH 7.4). Chicks were anesthetized by i.m. injection of 20–40 mg ketamine/kg weight and 3–5 mg xylazine/kg weight and perfused intracardially with 0.1 M sodium phosphate buffer (pH 7.4) followed by 4% paraformaldehyde in 0.1 M sodium phosphate buffer (pH 7.4).

### Cultures of dissociated retinal neurons and retinal explants

Six (E6) (HH 28–29) or seven days embryos (E7) (HH 30–31) were removed from the eggs, decapitated and their retinas were dissected out in ice-cold Hank's Balanced Salt Solution (HBSS).

The cornea, lens, marginal zone and vitreous humour were removed and discarded, exposing the neural retina which was separated from the retina pigmented epithelium. Retinas were divided into three sections from nasal to temporal pole, middle third was discarded and nasal or temporal thirds were employed.

For cultures of dissociated retinal neurons, the neural retinal portions were mechanically and enzymatically dissociated with a solution containing trypsin (0.1%), EDTA (1 mM) and DNAse (0.05%). The cells were resuspended in N2-supplemented (Invitrogen) F12/DMEM medium (Invitrogen) and cultured at 25,000 cells/cm^2^ on coated coverslips located onto culture dishes.

Retinal explants were obtained by cutting small pieces from nasal or temporal retinas, and cultured in N2-supplemented F12/DMEM containing 0.4% methylcellulose (Sigma) on coated coverslips located onto culture dishes.

In oder to evaluate the effect of soluble EphA3 ectodomain, substrates were prepared by incubating coverslips with poly-L-lysine (200 µg/ml) (Sigma) and laminin (20 µg/ml) (Invitrogen) for 2 hours at room temperature (RT) each one. To assay the effect substrate-bound EphA3 ectodomain, anti-human Fc polyclonal antibody (30 µg/ml) (Cappel), laminin (10 µg/ml) and human Fc (0.25 µg/ml = 7 nM) or EphA3-Fc at different concentrations (0.06 µg/ml = 0.5 nM to 1 µg/ml = 8 nM) (R&D Systems) were successively incubated 2 hours at RT each one [47].

Both the dissociated retinal neurons and retinal explants were cultured at 37°C and 5% CO2 for 24 hs. After it, the cultures were fixed with paraformaldehyde 2%-sacarose 2% in PBS for 30 minutes at RT.

### Stimulation with EphA3-Fc

EphA3-Fc fusion protein (R&D Systems) – the mouse EphA3 extracellular and transmembrane domain (Met1–His541) fused to Fc- was used to stimulate dissociated retinal neurons or retinal explants as substrate or as soluble clusters. Fc (R&D Systems) was used as control. In both cases EphA3-Fc and Fc were clustered with an anti-human Fc polyclonal antibody (Cappel). In order to evaluate the effect of substrate-bound EphA3-Fc, an anti-human Fc polyclonal antibody (30 µg/ml) (Cappel), laminin (10 µg/ml) and human Fc (0.25 µg/ml = 7 nM) or EphA3-Fc at different concentrations (0.06 µg/ml = 0.5 nM, 0.125 µg/ml = 1 nM, 0.25 µg/ml = 2 nM, 0.5 µg/ml = 4 nM, or 1 µg/ml = 8 nM) (R&D Systems) were successively incubated 2 hours at RT each one [47]. In order to evaluate the effect of soluble clustered EphA3-Fc, the anti-human Fc antibody and the EphA3-Fc or Fc were preincubated for 1 hour at RT, and then added to the N2-supplemented F12/DMEM where the dissociated retinal neurons had been seeded or to the N2-supplemented F12/DMEM containing 0.4% methylcellulose where the retinal explants had been plated [47]. After titration, 0.25 µg/ml (2 nM) of EphA3-Fc was used because it produced the maximal effect on nasal RGC axon growth.

### Time-lapse experiments

Culture dishes containing nasal or temporal explants were transferred to an inverted IX81 Olympus microscope (Tokyo, Japan) provided with a 37°C heated plate located in an incubation chamber with 5% CO_2_. Cultures were captured with phase-contrast imaging with a monitorized stage allowing for serial image adquisition. Cultures were automatically photographed each 3 minutes. Images were processed employing the NIH Image J software.

In order to evaluate the kinetics of axon growth, clustered EphA3-Fc or Fc were added to the medium and explants were followed for 18 hours from the beginning of the culture.

In order to evaluate the behavior of growth cones making contact with EphA3ΔC-EGFPN3 or EGFPF-stably transfected-HEK293 cells, HEK293 cells were cultured at a low confluence on a substrate of poly-L-lysine (200 µg/ml) and laminin (20 µg/ml) and then nasal or temporal retinal explants were added. After 12 hours, the cocultures were followed for 120 to 480 minutes.

HEK293 established commercial cell lines (human embryonic kidney epithelial cells) were obtained from ATCC.

### DNA construct and cell transfection

Chicken EphA3 cDNA was cut from pBluescript KS1 (Stratagen) expression vector employing ECORI and Asp718 restriction enzymes to obtain a trunkated EphA3 (bp 1 to 1728) without the cytoplasmic tyrosine kinase domain. This cDNA was introduced in pEGFPN3 N terminal protein fusion expression vector (Clontech) to obtain the fusion molecule EphA3ΔC-EGFPN3. HEK293 cells were transfected with EphA3ΔC-EGFPN3 or EGFPF employing SuperFect (Quiagen) and were subcloned using G418 to obtain stably-transfected cells.

### DNA construct and retrovirus production

EphA3ΔC-EGFPN3 and EGFPF were cloned into a NcoI/SmaI vector of the pCla12NCO helper plasmid, from which the corresponding ClaI fragment containing the 5′UTR of v-src was cloned into pRCAS-BP-B [57]. High-titers stocks of RCAS-BP-B-EphA3ΔC-EGFPN3 and RCAS-BP-B-EGFPF were generated by transfecting them into chicken embryo fibroblasts according to [58]. Assembled viruses were harvested and concentrated. All viral titers were >10^8^ IU/ml. Polybrene (Sigma) was added to the injection cocktail at 45 µg/ml and Fast Green (1^0^/_00_) was added to visualize the solution.

### EphA3 ectodomain misexpression and anterograde labeling of retinotectal projection

Eggs were windowed at E2 (HH13–16) and retroviral solution (RCAS-BP-B-EphA3ΔC-EGFPN3 or RCAS-BP-B-EGFPF) was injected into the tectum by using a micropipette with a Picospritzer II microinjector (Parker Hannifin Corp, New Yersey, USA). In ovo anterograde labeling of RGC axons was performed by local injection of 5 µl of DiI 282 (Invitrogen) (10^o^/_o_ w/v in ethanol) into the peripheral nasal or temporal retina between E9 (HH35) and E16 (HH42) [6]. Embryos were dissected and fixed with paraformaldehyde 4% in PBS between E11 (HH37) and E19 (HH45) (48 to 72 hours after DiI injection). Whole mounts of both the tecta and the retinas were prepared to detect the areas of RCAS-BP-B-EphA3ΔC-EGFPN3 or RCAS-BP-B-EGFPF expression and retinotectal projection. Some tecta were sectioned at 50 µm with a vibratome (World Precision Instruments Inc, Sarasota FL, USA) to determine the radial extension of the EGFP expression.

### Immunocytochemistry

Eyes and tecta were dissected out from chicken embryos every day between the 4^th^ (E4: HH23–24) and the 18^th^ days of incubation (E18; HH44), and from chicks between hatching and 7 postnatal days (P7). Chicken embryos were fixed in paraformaldehyde 4% in 0.1 M sodium phosphate buffer (pH 7.4) overnight at 4°C and chicks were fixed by perfusion with 4% paraformaldehyde in 0.1 M sodium phosphate buffer (pH 7.4) and the retinas and mesencephalon were post fixed for 3 hours in the same fixative at 4°C. They were cryoprotected by immersion in 25% sucrose in PBS overnight, embedded in Tissue-Tek O.C.T. Compound (Sakura Finetek), frozen in 2-methylbutane (Mallinckrodt Baker Inc.) and dry ice and stored at −20°C before sectioning with a cryostat (Leica CM 1900) at 25 µm, collected in gelatinized slides and stored at −20°C. All sections of optic tecta were performed along the rostro-caudal developmental gradient axis whereas all sections of retinas were performed along the naso-temporal axis. This allowed us to compare photographs obtained from different areas (rostral versus caudal tectum and nasal versus temporal retina) of the same section. Cultures were fixed with paraformaldehyde 2%-sacarose 2% in PBS for 30 minutes at RT and rinsed in PBS.

For immunofluorescence, nonspecific binding was blocked by preincubating in 5% normal goat serum (NGS) in PBS with or without 0.5% Tween 20 (Sigma) for 1 hour and then incubated with the primary antibodies. Cultures were incubated for 30 min at RT whereas sections were incubated overnight at 4°C. For double immunostaining, two antibodies were added at the same time. The following primary antibodies were used: rabbit anti-ephrin-A2 (L-20, sc912), anti-EphA3 (L-18, sc920) and anti-EphA4 (S-20, sc921) (Santa Cruz Biotech.), rabbit anti-ephrin-A5, anti-ephrin-A6, anti-EphA3 and anti-EphA4 (gifts from E.B. Pasquale, Sanford-Burnham Medical Research Institute, La Jolla, CA, USA) [12], [18], [19], rabbit anti-EphA4 (Tyr-602), phospho-specific antibody (Ep2731) (ECM Biosciences), mouse anti-EphA4 (D-4, sc 365503) (Santa Cruz Biotech.), and mouse anti-neuron specific unique β-tubulin (βIII) (MMS-435P, clone TUJ1) (COVANCE). They were diluted in PBS containing 2% NGS with or without 0.5% Tween 20 at 1–2 µg/ml. A negative control was done by omission of the primary antibody. Then they were incubated with Alexa Fluor 488 (green) or 594 (red)-conjugated F(ab')_2_ fragment of goat anti-rabbit antibody (A-11070, A-11072) (Molecular Probes) for primary polyclonal antibodies or with Alexa Fluor 488 or 594-conjugated F(ab')_2_ fragment of goat anti-mouse antibody (Molecular Probes)(A-11017, A-11020, Molecular Probes) for primary monoclonal antibodies, diluted 1∶2000 (1 µg/ml) in PBS containing 2% NGS for 2 hours at RT. Sections were then rinsed in PBS and some of them were counterstained by a 10 minutes incubation with the nuclear dye Hoechst 33342 (B-2261, Sigma) in PBS (1∶1000). They were mounted with Fluoromount-G (SouthernBiotech).

Actin cytoskeleton of RGC axons was labeled with Alexa Fluor 488-conjugated Phalloidin (A-12379, Molecular Probes). 5 µl of methanolic stock solution into 200 µl PBS was used for each coverslip for 20 minutes at RT.

### Photographs, quantification of axon outgrowth and interstitial filopodia, level of expression and colocalization

Retinal cultures, sections and whole mounts were observed under an Eclipse TS100 Nikon inverted microscopy (New York, USA) or a Zeiss Axiophot 2 microscopy (Oberkochen, Germany) with phase-contrast or epifluorescence. They were photographed with a Coolpix 4500 or an Axiocam HRC digital camera. Images were captured and reproduced in exactly the same form for all conditions in each experiment. Fluorescence intensity and distribution observed in sections were compared in different areas (nasal versus temporal retina or rostral versus caudal tectum) from the same structure in the same section. All compared areas were successively photographed with the same camera and the order of areas to be photographed was changed in different experiments, presenting the same difference in all cases. Images were assembled with PhotoShop software. Brightness and contrast were adjusted in the same form inside each experiment. Axonal and filopodia length were measured by using Image Pro Plus software. Axonal level of EphAs-ephrin-As expression was assayed by calculating the integral optic density (IOD) with NIH Image J software.

Confocal images were acquired on an Olympus FV300 microscope (Tokyo, Japan). The exposure settings and gain of laser were kept the same for all conditions in each experiment. Ten fields were acquired by condition, and a single focal plane by field was used for comparison. Image analysis, algorithm generation and statistical analysis were performed under Image Pro Plus 6.0. Before evaluating colocalization of two stainings in paired images, the relevant “regions of interest” (ROIs) were determined [59] in both images in order to separate signal from background but also to determine a common region for both images in which putative signal fluctuations might be relevant. For the results to be compared, all background correction settings were the same for all images in a study. Pearson's correlation coefficient (values indicating colocalization: from 0.5 to 1.0). and Manders overlap coefficient (values indicating colocalization: from 0.6 to 1.0) [60] were evaluated in order to establish the level of colocalization and perform comparisons. We found similar results by using both coefficients of colocalization.

### Immunoblotting

Samples obtained from E8 nasal and temporal retina were homogenized in buffer lysis [TRIS 50 mM buffer containing EDTA 1 mM, ClNa 150 mM (pH:8), proteases inhibitor cocktail (1/100) (Sigma) and NaVo3 100 mM]. They were centrifuged 10 min at 1000 g at 4°C and the supernatant was centrifuged 20 min at 20000 g at 4°C. The second pellet was used after to be resuspended in buffer lysis with octyl beta-D-glucopyranoside 1% (Calbiochem) and Triton X100 0.5%. Protein concentrations were measured using a Pierce BCA protein assay kit. Equal amounts of protein were loaded in each lane. Proteins were separated by SDS-PAGE 10% and transferred onto a PVDF filter. Filters were blocked in 4% NGS in TTBS for 1 hour and incubated overnight in 1% NGS in TTBS containing rabbit anti-EphA4 (Tyr-602), phospho-specific (Ep2731) (ECM Biosciences) (1/1000) or anti-ephrin-A2 antibodies (L-20, sc912) (Santa Cruz Biotech) (1/1000). After washes filters were incubated for 2 hours with 1% NGS in TTBS containing HRP-conjugated goat anti-mouse IgG-HRP (sc-2005) or goat anti-rabbit IgG-HRP (sc-2004) (Santa Cruz Biotech) (1/1000). Filters were developed with chemoluminescent substrate (Pierce ECL). They were stripped and reprobed with a rabbit anti-EphA4 antibody (S-20, sc921) (1/1000) or a mouse anti-actin antibody (2Q1055, sc58673) (Santa Cruz Biotech.) (1/5000) (load control). Quantification of Tyr-602-phosphorylated EphA4, total EphA4, ephrin-A2, actin and their ratios was made by measuring the bands density with Gel Pro software.

### Statistical analysis

All the quantifications were made in blind to experimental conditions. Data were expressed as mean +/− SE. ANOVA and Tukey postest or Student's t test were used for comparisons of axon length and interstitial filopodia density, intensity of labeling in immunocytochemistry and in bands of Westernblots, proportions of labeled axons and proportions of passing axons and termination zones. Non parametric Mann Whitney test was employed when samples did not present normal distributions. Fisher's exact test was used to compare proportions of growth cones with different behaviors in time-lapse experiments. p<0.05 was considered significant.

### Developmental optic tectum: Layers Nomenclature

In order to describe the developmental expression of EphA3 in the chicken optic tectum we used a nomenclature that describes the tectal lamination as a dynamic process of transient cell compartments segregation and establishes tectal developmental stages according to the lamination process [1], [3]. The designation of the embryonic lamina as “transient cell compartment” (TCC) emphasizes the fact that they are not precursors of particular adult layers, but transient aggregations or mixing of neurons that will later segregate into several different definitive layers. As development progresses, an early TCC segregates into subsequent TCCs until particular neuronal populations reach their specific postmigratory sites. In this nomenclature, the different TCCs that successively appear during development are designated with a number that refers to their chronological order of appearance. The embryonic laminae are designated with a more specific name only when they can be topographically identified as precursors of particular definitive layers. However, along the entire migratory phase the embryonic laminae are named as “compartments” (C) instead of layers because their cell composition does not necessarily coincide with that of the definitive layers. From the end of the migratory process onwards, the nomenclature for the adult tectum can be properly used [61].

## Supporting Information

Figure S1 **Anterograde labeling of RGC axons in the retina and EphA3 ectodomain overexpression in the tectum.** (A) After DiI labeling at E11 (HH37), the retina was analyzed in a whole mount at E13 (HH39). Photograph montage shows RGCs labeled with DiI. Arrows depict the local injection area (LIn) from which retrogradely labeled axons (OF) show the RGCs somas (S) and anterogradely labeled axons (OF) form two fascicles. Scale bars: 200 µm. (B, C) After infection of the optic tectum at E2 (HH14–15) with RCAS-BP-B-EphA3ΔC-EGFP and DiI labeling of the naso-dorsal retina at E11, the tectum was analyzed in vibratome sections obtained along its rostro-caudal axis at E13. EGFP expression depicts tectal cells which overexpress EphA3ΔC-EGFP (LI). They form columnar arrangements along all the tectal radial extension. Pial surface is upper and ventricular surface is at the bottom. An optic fiber (OF) grows in the stratum opticum (SO) from rostral to caudal tectum in B. Scale bars: 100 µm and 50 µm respectively.
(TIF)

Video S1 **Related to** **Fig. 4C** **. Behavior of nasal RGC axon growth cones making contact with EGFPF-transfected-HEK293 cells.** Time-lapse experiment showed as movie. A nasal growth cone indistinctly attaches and retracts from EGFPF-transfected-HEK293 cells (control). Total time: 147 minutes, interval per photograph: 3 minutes.
(MOV)

Video S2 **Related to** **Fig. 4D** **. Behavior of nasal RGC axon growth cones making contact with EphA3ΔC-EGFP-transfected-HEK293 cells.** Time-lapse experiment showed as movie. Nasal growth cones grow along EphA3ΔC-EGFP-transfected-HEK293 cells. Total time: 183 minutes, interval per photograph: 3 minutes.
(MOV)

Video S3 **Related to** **Fig. 4E** **. Behavior of temporal RGC axon growth cones making contact with EphA3ΔC-EGFP-transfected-HEK293 cells.** Time-lapse experiment showed as movie. A temporal growth cone adheres to EphA3ΔC-EGFP-transfected-HEK293 cells. Total time: 471 minutes, interval per photograph: 3 minutes. Each video is presented successively: a) without signals, b) with a circle depicting the growth cones, and c) with a circle depicting the growth cones and the surrounding surface in a darker grey level to highlight the growth cones inside the circle.
(MOV)

## References

[1] Scicolone G, Ortalli AL, Carri NG (2009). Key roles of Ephs and ephrins in retinotectal topographic map formation.. Brain Res Bull.

[2] Feldheim DA, O'Leary DD (2010). Visual map development: bidirectional signaling, bifunctional guidance molecules, and competition.. Cold Spring Harb Perspect Biol.

[3] Rapacioli M, Rodriguez Celin A, Duarte S, Ortalli AL, Di Napoli J (2011). The chick optic tectum developmental stages. A dynamic table based on temporal- and spatial-dependent histogenetic changes: A structural, morphometric and immunocytochemical analysis.. J Morphol.

[4] Scicolone G, Ortalli AL, Alvarez G, Lopez-Costa JJ, Rapacioli M (2006). Developmental pattern of NADPH-diaphorase positive neurons in chick optic tectum is sensitive to changes in visual stimulation.. J Comp Neurol.

[5] Scicolone G, Pereyra-Alfonso S, Brusco A, Pecci Saavedra J, Flores V (1995). Development of the laminated pattern of the chick tectum opticum.. Int J Dev Neurosci.

[6] Yates PA, Roskies AL, McLaughlin T, O'Leary DD (2001). Topographic-specific axon branching controlled by ephrin-As is the critical event in retinotectal map development.. J Neurosci.

[7] Sperry RW (1963). Chemoaffinity in the Orderly Growth of Nerve Fiber Patterns and Connections.. Proc Natl Acad Sci U S A.

[8] Gebhardt C, Bastmeyer M, Weth F (2012). Balancing of ephrin/Eph forward and reverse signaling as the driving force of adaptive topographic mapping.. Development.

[9] Bevins N, Lemke G, Reber M (2011). Genetic Dissection of EphA Receptor Signaling Dynamics during Retinotopic Mapping.. J Neurosci.

[10] Reber M, Burrola P, Lemke G (2004). A relative signalling model for the formation of a topographic neural map.. Nature.

[11] Pfeiffenberger C, Yamada J, Feldheim DA (2006). Ephrin-As and patterned retinal activity act together in the development of topographic maps in the primary visual system.. J Neurosci.

[12] Simpson HD, Mortimer D, Goodhill GJ (2009). Theoretical models of neural circuit development.. Curr Top Dev Biol.

[13] Tsigankov D, Koulakov AA (2010). Sperry versus Hebb: topographic mapping in Isl2/EphA3 mutant mice.. BMC Neurosci.

[14] Flanagan JG (2006). Neural map specification by gradients.. Curr Opin Neurobiol.

[15] McLaughlin T, O'Leary DD (2005). Molecular gradients and development of retinotopic maps.. Annu Rev Neurosci.

[16] Pasquale EB (2005). Eph receptor signalling casts a wide net on cell behaviour.. Nat Rev Mol Cell Biol.

[17] Vearing CJ, Lackmann M (2005). “Eph receptor signalling; dimerisation just isn't enough”.. Growth Factors.

[18] Connor RJ, Menzel P, Pasquale EB (1998). Expression and tyrosine phosphorylation of Eph receptors suggest multiple mechanisms in patterning of the visual system.. Dev Biol.

[19] Menzel P, Valencia F, Godement P, Dodelet VC, Pasquale EB (2001). Ephrin-A6, a new ligand for EphA receptors in the developing visual system.. Dev Biol.

[20] Brown A, Yates PA, Burrola P, Ortuno D, Vaidya A (2000). Topographic mapping from the retina to the midbrain is controlled by relative but not absolute levels of EphA receptor signaling.. Cell.

[21] Carreres MI, Escalante A, Murillo B, Chauvin G, Gaspar P (2011). Transcription factor Foxd1 is required for the specification of the temporal retina in mammals.. J Neurosci.

[22] Park S, Frisen J, Barbacid M (1997). Aberrant axonal projections in mice lacking EphA8 (Eek) tyrosine protein kinase receptors.. Embo J.

[23] Marin O, Blanco MJ, Nieto MA (2001). Differential expression of Eph receptors and ephrins correlates with the formation of topographic projections in primary and secondary visual circuits of the embryonic chick forebrain.. Dev Biol.

[24] Rashid T, Upton AL, Blentic A, Ciossek T, Knoll B (2005). Opposing gradients of ephrin-As and EphA7 in the superior colliculus are essential for topographic mapping in the mammalian visual system.. Neuron.

[25] Hornberger MR, Dutting D, Ciossek T, Yamada T, Handwerker C (1999). Modulation of EphA receptor function by coexpressed ephrinA ligands on retinal ganglion cell axons.. Neuron.

[26] Monschau B, Kremoser C, Ohta K, Tanaka H, Kaneko T (1997). Shared and distinct functions of RAGS and ELF-1 in guiding retinal axons.. Embo J.

[27] Cheng HJ, Nakamoto M, Bergemann AD, Flanagan JG (1995). Complementary gradients in expression and binding of ELF-1 and Mek4 in development of the topographic retinotectal projection map.. Cell.

[28] Drescher U, Kremoser C, Handwerker C, Loschinger J, Noda M (1995). In vitro guidance of retinal ganglion cell axons by RAGS, a 25 kDa tectal protein related to ligands for Eph receptor tyrosine kinases.. Cell.

[29] Feldheim DA, Kim YI, Bergemann AD, Frisen J, Barbacid M (2000). Genetic analysis of ephrin-A2 and ephrin-A5 shows their requirement in multiple aspects of retinocollicular mapping.. Neuron.

[30] Nakamoto M, Cheng HJ, Friedman GC, McLaughlin T, Hansen MJ (1996). Topographically specific effects of ELF-1 on retinal axon guidance in vitro and retinal axon mapping in vivo.. Cell.

[31] Sakurai T, Wong E, Drescher U, Tanaka H, Jay DG (2002). Ephrin-A5 restricts topographically specific arborization in the chick retinotectal projection in vivo.. Proc Natl Acad Sci U S A.

[32] Feldheim DA, Nakamoto M, Osterfield M, Gale NW, DeChiara TM (2004). Loss-of-function analysis of EphA receptors in retinotectal mapping.. J Neurosci.

[33] Carvalho RF, Beutler M, Marler KJ, Knoll B, Becker-Barroso E (2006). Silencing of EphA3 through a cis interaction with ephrinA5.. Nat Neurosci.

[34] Dutting D, Handwerker C, Drescher U (1999). Topographic targeting and pathfinding errors of retinal axons following overexpression of ephrinA ligands on retinal ganglion cell axons.. Dev Biol.

[35] Gosse NJ, Nevin LM, Baier H (2008). Retinotopic order in the absence of axon competition.. Nature.

[36] Lim YS, McLaughlin T, Sung TC, Santiago A, Lee KF (2008). p75 (NTR) mediates ephrin-A reverse signaling required for axon repulsion and mapping.. Neuron.

[37] Marler KJ, Becker-Barroso E, Martinez A, Llovera M, Wentzel C (2008). A TrkB/EphrinA interaction controls retinal axon branching and synaptogenesis.. J Neurosci.

[38] Marler KJ, Poopalasundaram S, Broom ER, Wentzel C, Drescher U (2010). Pro-neurotrophins secreted from retinal ganglion cell axons are necessary for ephrinA-p75NTR-mediated axon guidance.. Neural Dev.

[39] Poopalasundaram S, Marler KJ, Drescher U (2011). EphrinA6 on chick retinal axons is a key component for p75(NTR)-dependent axon repulsion and TrkB-dependent axon branching.. Mol Cell Neurosci.

[40] McLaughlin T, Hindges R, O'Leary DD (2003). Regulation of axial patterning of the retina and its topographic mapping in the brain.. Curr Opin Neurobiol.

[41] Herrera E, Marcus R, Li S, Williams SE, Erskine L (2004). Foxd1 is required for proper formation of the optic chiasm.. Development.

[42] Takahashi H, Sakuta H, Shintani T, Noda M (2009). Functional mode of FoxD1/CBF2 for the establishment of temporal retinal specificity in the developing chick retina.. Dev Biol.

[43] Takahashi H, Shintani T, Sakuta H, Noda M (2003). CBF1 controls the retinotectal topographical map along the anteroposterior axis through multiple mechanisms.. Development.

[44] Muhleisen TW, Agoston Z, Schulte D (2006). Retroviral misexpression of cVax disturbs retinal ganglion cell axon fasciculation and intraretinal pathfinding in vivo and guidance of nasal ganglion cell axons in vivo.. Dev Biol.

[45] Cohen J, Nurcombe V, Jeffrey P, Edgar D (1989). Developmental loss of functional laminin receptors on retinal ganglion cells is regulated by their target tissue, the optic tectum.. Development.

[46] Halfter W, Claviez M, Schwarz U (1981). Preferential adhesion of tectal membranes to anterior embryonic chick retina neurites.. Nature.

[47] Wang HU, Anderson DJ (1997). Eph family transmembrane ligands can mediate repulsive guidance of trunk neural crest migration and motor axon outgrowth.. Neuron.

[48] Davenport RW, Thies E, Cohen ML (1999). Neuronal growth cone collapse triggers lateral extensions along trailing axons.. Nat Neurosci.

[49] Dent EW, Kalil K (2001). Axon branching requires interactions between dynamic microtubules and actin filaments.. J Neurosci.

[50] Bonhoeffer F, Huf J (1985). Position-dependent properties of retinal axons and their growth cones.. Nature.

[51] Kao TJ, Kania A (2011). Ephrin-Mediated cis-Attenuation of Eph Receptor Signaling Is Essential for Spinal Motor Axon Guidance.. Neuron.

[52] Yoo S, Kim Y, Noh H, Lee H, Park E (2011). Endocytosis of EphA receptors is essential for the proper development of the retinocollicular topographic map.. Embo J.

[53] Brunet I, Weinl C, Piper M, Trembleau A, Volovitch M (2005). The transcription factor Engrailed-2 guides retinal axons.. Nature.

[54] Wizenmann A, Brunet I, Lam JS, Sonnier L, Beurdeley M (2009). Extracellular Engrailed participates in the topographic guidance of retinal axons in vivo.. Neuron.

[55] Matsunaga E, Nakamura H, Chedotal A (2006). Repulsive guidance molecule plays multiple roles in neuronal differentiation and axon guidance.. J Neurosci.

[56] Hamburger V, Hamilton H (1951). A series of normal stages in the development of the chick embryo..

[57] Hughes SH, Greenhouse JJ, Petropoulos CJ, Sutrave P (1987). Adaptor plasmids simplify the insertion of foreign DNA into helper-independent retroviral vectors.. J Virol.

[58] Fekete DM, Cepko CL (1993). Replication-competent retroviral vectors encoding alkaline phosphatase reveal spatial restriction of viral gene expression/transduction in the chick embryo.. Mol Cell Biol.

[59] Jaskolski F, Mulle C, Manzoni OJ (2005). An automated method to quantify and visualize colocalized fluorescent signals.. J Neurosci Methods.

[60] Zinchuk V, Grossenbacher-Zinchuk O (2009). Recent advances in quantitative colocalization analysis: focus on neuroscience.. Prog Histochem Cytochem.

[61] Cowan WM, Adamson L, Powell TP (1961). An experimental study of the avian visual system.. J Anat.

