# Supplementary figures and images for: EphA3 Expressed in the Chicken Tectum Stimulates Nasal Retinal Ganglion Cell Axon Growth and Is Required for Retinotectal Topographic Map Formation
Source: PLoS One. 2012 Jun 7;7(6):e38566. doi: 10.1371/journal.pone.0038566 (PMC3369860; doi:10.1371/journal.pone.0038566)

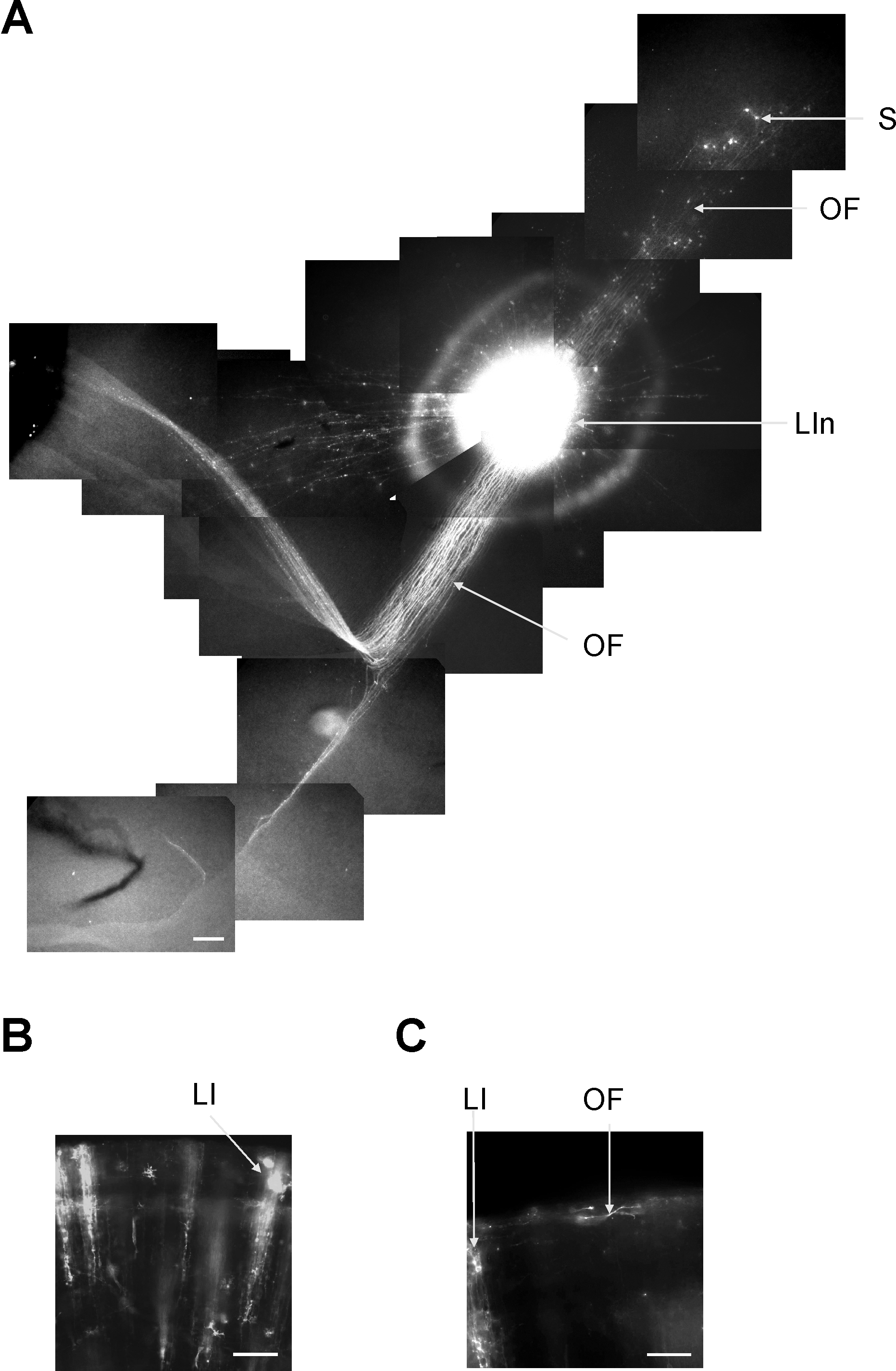

Supplement: Figure S1 — Anterograde labeling of RGC axons in the retina and EphA3 ectodomain overexpression in the tectum. (A) After DiI labeling at E11 (HH37), the retina was analyzed in a whole mount at E13 (HH39). Photograph montage shows RGCs labeled with DiI. Arrows depict the local injection area (LIn) from which retrogradely labeled axons (OF) show the RGCs somas (S) and anterogradely labeled axons (OF) form two fascicles. Scale bars: 200 µm. (B, C) After infection of the optic tectum at E2 (HH14–15) with RCAS-BP-B-EphA3ΔC-EGFP and DiI labeling of the naso-dorsal retina at E11, the tectum was analyzed in vibratome sections obtained along its rostro-caudal axis at E13. EGFP expression depicts tectal cells which overexpress EphA3ΔC-EGFP (LI). They form columnar arrangements along all the tectal radial extension. Pial surface is upper and ventricular surface is at the bottom. An optic fiber (OF) grows in the stratum opticum (SO) from rostral to caudal tectum in B. Scale bars: 100 µm and 50 µm respectively. (TIF) [file pone.0038566.s001.tif]
